# The transcriptome of HIV-1 infected intestinal CD4+ T cells exposed to enteric bacteria

**DOI:** 10.1371/journal.ppat.1006226

**Published:** 2017-02-27

**Authors:** Alyson C. Yoder, Kejun Guo, Stephanie M. Dillon, Tzu Phang, Eric J. Lee, Michael S. Harper, Karen Helm, John C. Kappes, Christina Ochsenbauer, Martin D. McCarter, Cara C. Wilson, Mario L. Santiago

**Affiliations:** 1 Department of Medicine, University of Colorado School of Medicine, Aurora, CO, United States of America; 2 RNA Bioscience Initiative, University of Colorado School of Medicine, Aurora, CO, United States of America; 3 The Cancer Center, University of Colorado School of Medicine, Aurora, CO, United States of America; 4 Department of Immunology and Microbiology, University of Colorado School of Medicine, Aurora, CO, United States of America; 5 Department of Medicine, University of Alabama at Birmingham, Birmingham, AL, United States of America; 6 Center for AIDS Research, University of Alabama at Birmingham, Birmingham, AL, United States of America; 7 Department of Surgery, University of Colorado School of Medicine, Aurora, CO, United States of America; Miller School of Medicine, UNITED STATES

## Abstract

Global transcriptome studies can help pinpoint key cellular pathways exploited by viruses to replicate and cause pathogenesis. Previous data showed that laboratory-adapted HIV-1 triggers significant gene expression changes in CD4+ T cell lines and mitogen-activated CD4+ T cells from peripheral blood. However, HIV-1 primarily targets mucosal compartments during acute infection *in vivo*. Moreover, early HIV-1 infection causes extensive depletion of CD4+ T cells in the gastrointestinal tract that herald persistent inflammation due to the translocation of enteric microbes to the systemic circulation. Here, we profiled the transcriptome of primary intestinal CD4+ T cells infected *ex vivo* with transmitted/founder (TF) HIV-1. Infections were performed in the presence or absence of *Prevotella stercorea*, a gut microbe enriched in the mucosa of HIV-1-infected individuals that enhanced both TF HIV-1 replication and CD4+ T cell death *ex vivo*. In the absence of bacteria, HIV-1 triggered a cellular shutdown response involving the downregulation of HIV-1 reactome genes, while perturbing genes linked to OX40, PPAR and FOXO3 signaling. However, in the presence of bacteria, HIV-1 did not perturb these gene sets or pathways. Instead, HIV-1 enhanced granzyme expression and Th17 cell function, inhibited G1/S cell cycle checkpoint genes and triggered downstream cell death pathways in microbe-exposed gut CD4+ T cells. To gain insights on these differential effects, we profiled the gene expression landscape of HIV-1-uninfected gut CD4+ T cells exposed to bacteria. Microbial exposure upregulated genes involved in cellular proliferation, MAPK activation, Th17 cell differentiation and type I interferon signaling. Our findings reveal that microbial exposure influenced how HIV-1 altered the gut CD4+ T cell transcriptome, with potential consequences for HIV-1 susceptibility, cell survival and inflammation. The HIV-1- and microbe-altered pathways unraveled here may serve as a molecular blueprint to gain basic insights in mucosal HIV-1 pathogenesis.

## Author summary

The gastrointestinal (GI) tract is a major site of early HIV-1 replication and death of CD4+ T cells. As HIV-1 replicates in the gut, the protective epithelial barrier gets disrupted, causing the entry of bacteria into the underlying tissue and the bloodstream, leading to inflammation and clinical complications even in HIV-1-infected patients taking antiviral drugs. Counteracting these pathogenic processes may require in-depth understanding of the molecular pathways that HIV-1 and microbes utilize to infect, functionally alter and/or kill CD4+ T cells. However, to date, the nature of the genes altered by transmitted/founder HIV-1 strains and gut bacteria in intestinal CD4+ T cells remains unclear. Here, we obtained the first gene expression profiles of primary gut CD4+ T cells infected in cell culture with HIV-1 in the context of microbes found in the GI tract of HIV-1 infected patients. Our findings reveal common and distinct signaling pathways altered by HIV-1 depending on the presence of microbes that may shed light on infection, inflammation and CD4+ T cell depletion in HIV-1 infected individuals. More detailed studies of these molecular programs may inform potential ways to counteract pathogenic outcomes initiated and/or sustained by HIV-1 infection in the GI tract.

## Introduction

CD4+ T cells are the major targets of HIV-1 infection, and their preferential depletion during the course of infection is the hallmark feature of progression to AIDS [1]. Thus, major efforts have been made to understand the molecular events that occur following HIV-1 infection of CD4+ T cells. Insights on the cellular pathways inhibited and/or hijacked by HIV-1 have been obtained through global transcriptome profiling. Microarray studies reported that infection of CD4+ T cells with laboratory-adapted, CXCR4-tropic HIV-1 altered pathways associated with DNA repair, T cell activation, cell cycle control, subcellular trafficking, programmed cell death, RNA processing and nucleic acid metabolism (reviewed in [2]). However, those studies used either CD4+ T cell lines or mitogen-activated CD4+ T cells from peripheral blood, which are resistant to killing by primary, CCR5-tropic HIV-1 strains *in vitro* [3]. To date, the CD4+ T cell-intrinsic pathways altered by transmitted/founder (TF) HIV-1, which best approximate the initial strains, i.e. those identified to have established clinical infection *in vivo* [4, 5], remain unknown.

Regardless of the route of transmission, acute HIV-1 infection is characterized by high levels of replication and CD4+ T cell depletion in the gastrointestinal (GI) tract [6–8]. The GI tract harbors large numbers of activated memory CD4+ T cells expressing CCR5 [9], the coreceptor used by nearly all TF HIV-1 strains [10]. Within the first year of HIV-1 infection, preferential depletion of gut CD4+ T cell subsets that produce IL17 (Th17) and IL22 (Th22) were documented [11, 12]. Th17 and Th22 cells protect the integrity of the epithelial barrier, and their selective depletion has been linked to gut barrier disruption and the translocation of enteric commensal microbes to the systemic circulation [13–15]. This phenomenon, referred to as ‘microbial translocation’, is now widely accepted as a fundamental mechanism driving HIV-1-associated chronic immune activation. Notably, a microarray study using intestinal mucosal biopsies from patients 4 to 8 weeks following HIV-1 infection revealed the upregulation of interferon (IFN), immune activation, inflammation, chemotaxis, cell cycle and apoptotic pathways compared to HIV-1 uninfected patients [16]. These findings revealed that early HIV-1 infection altered host gene expression in the GI tract *in vivo*. However, it remains unclear which triggers (viral or bacterial) and cell types [e.g., T cells, dendritic cells (DCs), epithelial cells] in the bulk tissues were driving these gene expression changes.

To model how the interactions between intestinal lamina propria mononuclear cells (LPMCs), CCR5-tropic HIV-1 and the microbiota influence CD4+ T cell death, we developed the Lamina Propria Aggregate Culture (LPAC) model [17, 18]. In contrast to HIV-1 infection of PBMCs, the LPAC model does not require prior exogenous mitogen stimulation to obtain reproducible CCR5-tropic HIV-1 infection and CD4+ T cell death. Using the LPAC model, we previously showed that exposure of LPMCs to gut microbes enriched in the intestinal mucosa of HIV-1-infected patients [19, 20] enhanced HIV-1 infection and CD4+ T cell death [20]. Thus, the LPAC model provides a unique opportunity to catalogue the gene profile of a gut CD4+ T cell infected with TF HIV-1 in the presence or absence of enteric microbes. These altered gene signatures and pathways may in turn provide novel avenues for gaining basic insights on mucosal HIV-1 pathogenesis.

## Results

### Transmitted/Founder (TF) HIV-1 strains kill lamina propria (LP) CD4+ T cells *ex vivo*

**Fig 1:**
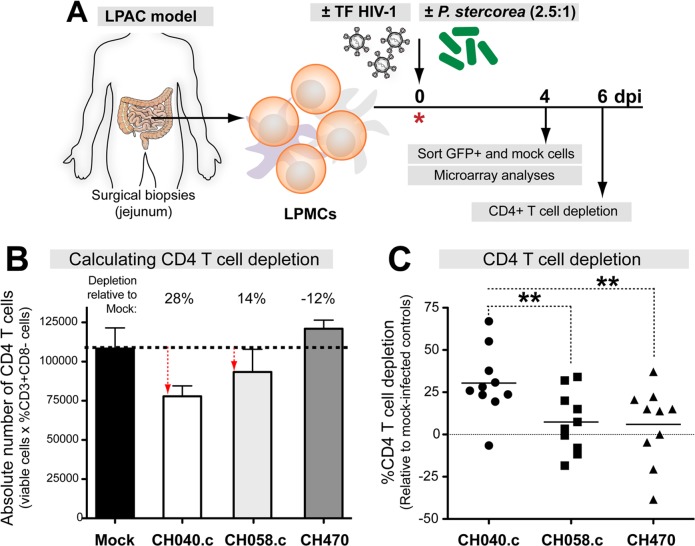
TF HIV-1 strains deplete CD4+ T cells in the LPAC model. (A) LPAC model. LPMCs were obtained from jejunum biopsies of HIV-1-uninfected individuals and used to infect with p24-normalized TF HIV-1 strains in the presence or absence of *Prevotella stercorea*. CD4+ T cells were analyzed at 6 days post-infection (dpi). (B) Depletion values were computed by comparing the mean absolute numbers of CD3+CD8- cells in triplicate HIV-1 infected versus mock cultures. Negative depletion values can be oftained if the CD3+CD8- cells in HIV-1-infected were greater than mock. (C) %CD4+ T cell killing data were compiled from multiple donors. Lines correspond to means and statistical analyses were performed using a one-way ANOVA followed by a Dunnett’s multiple comparison test. **p<0.01; ***p<0.001; ns, not significant.

Modeling early events in primary HIV-1 infection and depletion in the gut *ex vivo* may require the use of relevant HIV-1 strains. In previous studies with the LPAC model, we utilized a laboratory adapted R5-tropic HIV-1 strain, Ba-L [17, 18, 20]. To determine the nature of HIV-1 strains that initiated and established clinical infection in patients, TF HIV-1 sequences were inferred using a phylogenetic model of acute HIV-1 infection sequences [5, 10]. To investigate if TF HIV-1 strains caused LP CD4+ T cell death, we spinoculated LPMCs (n = 9–11 donors) with normalized levels of TF HIV-1 strains CH058.c, CH470 and CH040.c (Fig 1A). At 6 days post infection (dpi), absolute CD4+ T cell counts were calculated by flow cytometry and automated cell counting relative to mock controls (Fig 1B). Infection with CH040.c resulted in detectable CD4+ T cell depletion relative to mock-infected cells in 90% of LPMC donors, whereas CH058.c and CH470 depleted CD4+ T cells in 60% of LPMC donors by 6 dpi (Fig 1C). The differences in CD4+ T cell killing potential between TF HIV-1 strains suggest that viral factors may contribute to CD4+ T cell death in the LPAC model. Due to consistent depletion at 6 dpi, we chose HIV-1 CH040.c TF strain for subsequent global transcriptome profiling.

### HIV-1 infected gut CD4+ T cells display a distinct transcriptome profile

Transcriptome data on HIV-1 infection remains limited to CD4+ T cell lines, mitogen-activated CD4+ T cells and bulk tissue samples from HIV-1-infected individuals [2]. CD4+ T cells productively infected with HIV-1 can be evaluated by intracellular HIV-1 p24 flow cytometry, but this method requires a membrane permeabilization step that could compromise transcript levels. To capture live, intact CD4+ T cells productively infected with TF HIV-1, we constructed an HIV-1 CH040.c infectious molecular clone encoding eGFP, referred to herein as CH040.c-eGFP. The eGFP insert utilized the start site of *nef*, followed by a T2A self-cleaving peptide, then *nef*. As expected, high proportions of CD4+ T cells expressing eGFP (>80%) were positive for intracellular HIV-1 p24 by flow cytometry (S1 Fig).

LPMCs from 4 donors were spinoculated with CH040.c-eGFP for 2 h. As controls, untransfected 293T cell supernatants were used for mock infection (Fig 1A). Since HIV-1 downregulates CD4, viable, infected CD4+ T cells were sorted as CD3+CD8-GFP+ at 4 dpi using the gating strategy shown (S2 Fig). On average, ~1% of CD3+CD8- cells were GFP+ after 4 days of CH040.c-eGFP infection of LPMCs. Thus, large numbers of LPMCs (15 million) had to be infected to ensure that sufficient numbers of HIV-1 CH40 GFP+ cells were sorted for microarray analyses. We successfully sorted 5,660 to 26,171 viable GFP+ cells, yielding 72 to 360 pg of RNA, which was sufficient for microarray analyses but not enough for multiple quantitative PCR analyses. Viable CD3+CD8- cells were also sorted from mock infected LPMCs. Total RNA isolated from sorted cells was amplified and labeled using the WT Pico kit (Qiagen) for transcriptome profiling using Affymetrix Human Gene 2.0 arrays. These arrays cover >30,000 coding transcripts and >11,000 long intergenic non-coding transcripts.

**Fig 2:**
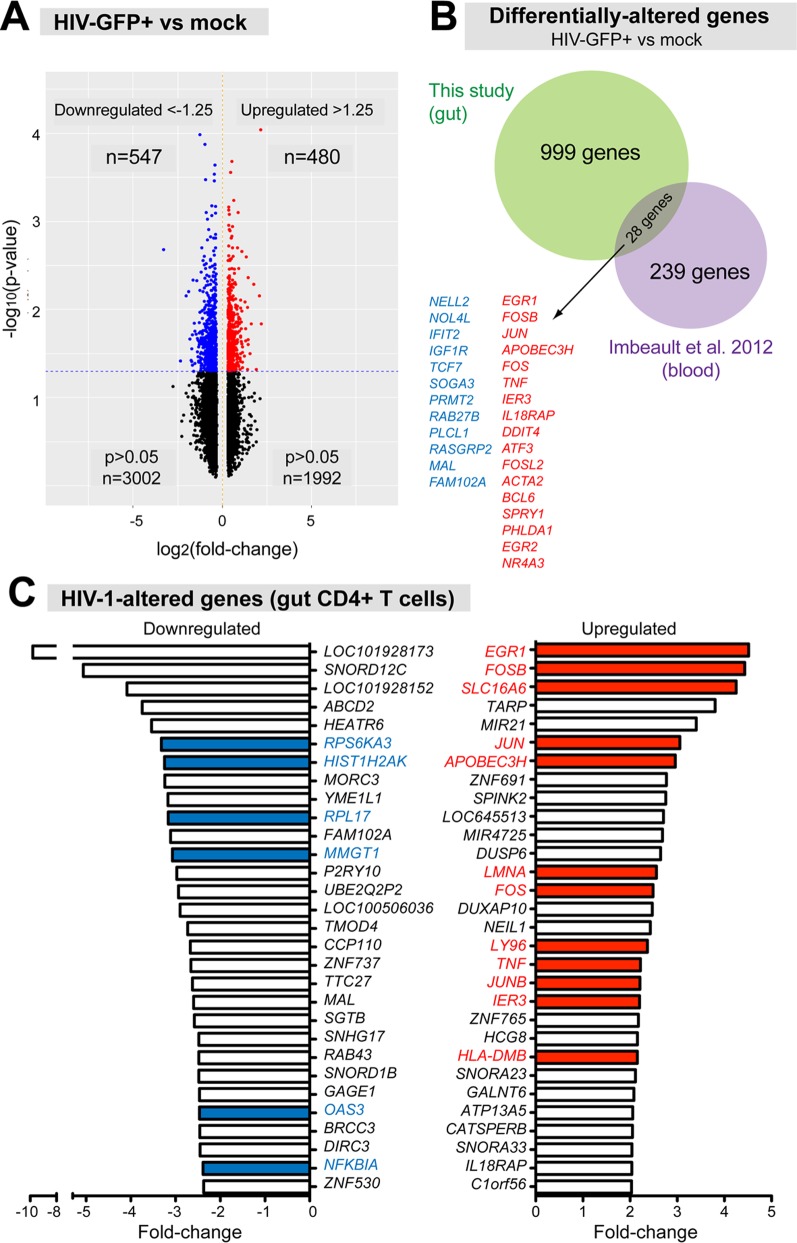
HIV-1 infection alters the gene expression profile of gut CD4+ T cells. LPMCs from 4 different donors were infected with CH040.c-eGFP and at 4 dpi, GFP+ and matched mock-infected CD4+ T cells were sorted for microarray analyses. Paired t-statistics were performed to determine altered genes. (A) Volcano plots showing altered genes in gut CD4+ T cells following HIV-1 infection, based on a 1.25-fold change cut-off and p<0.05. (B) Comparison of HIV-1-altered genes between this study and a published study using X4-tropic HIV-1 on mitogen-activated peripheral blood CD4+ T cells [21]. Genes in blue were downregulated; genes in red were upregulated. (C) Top 30 downregulated and upregulated genes due to HIV-1 infection, based on fold-change values. Colored bars/genes were previously shown to be involved in HIV-1 infection, see S2 Table for more details.

Principal component analysis of the full gene expression data revealed strong donor dependence (S3A Fig), consistent with the heterogeneity of LPMCs from diverse donors. To identify genes consistently altered in the 4 LPMC donors, we utilized two criteria: a *p*-value of <0.05 using paired t-statistics and a fold-change cut-off of 1.25 (S3B Fig). Using these criteria, we detected 1,207 genes that were altered in HIV-1 infected (GFP+) versus uninfected (mock) LP CD4+ T cells (Fig 2A). To determine if these genes were novel, we compared our dataset of differentially regulated genes to a recent microarray study using peripheral blood CD4+ T cells [21]. In this report, X4-tropic HIV-1 genetically linked to heat-stable antigen (HSA) was used to infect phytohemagglutinin-activated blood CD4 T cells that were co-cultured with IL-2. HSA+ cells were enriched by magnetic bead selection and analyzed by microarray. The authors found 267 differentially-regulated genes using a 1.7-fold cut-off. Notably, we found that only 3% of differentially expressed genes from our dataset overlapped with the Imbeault *et al* study [21] (Fig 2B), even though we utilized a lower fold-change cut-off value. These genes include *ATF3*, *FOSL2*, *JUN*, *FOS* and *FOSB* from the *FOS/JUN* gene family (Fig 2B; S1 Table). Thus, the majority of differentially-regulated genes due to HIV-1 infection in gut CD4+ T cells were novel relative to the Imbeault *et al* study. Fig 2C, S4 Fig and S2 Table lists the top-ranked and full list of upregulated and downregulated genes due to HIV-1 infection. Many of the genes were previously reported to be involved in HIV-1 replication. Our data suggests that HIV-1 induces a distinct transcriptome profile in primary gut CD4+ T cells compared to mitogen-activated blood CD4+ T cells.

### HIV-1 infection downregulated HIV-1 reactome gene sets

To determine enriched biological themes in HIV GFP+ versus mock-infected cells, we conducted gene set enrichment analysis (GSEA). GSEA is a computational method that determines whether a previously defined gene set shows statistically significant differences between two biological states [22]. Gene lists pre-ranked according to t-statistics allows comparisons across array platforms through calculation of enrichment scores, which reflects the degree to which a gene set is overrepresented at the top or bottom of a ranked list of genes.

**Fig 3:**
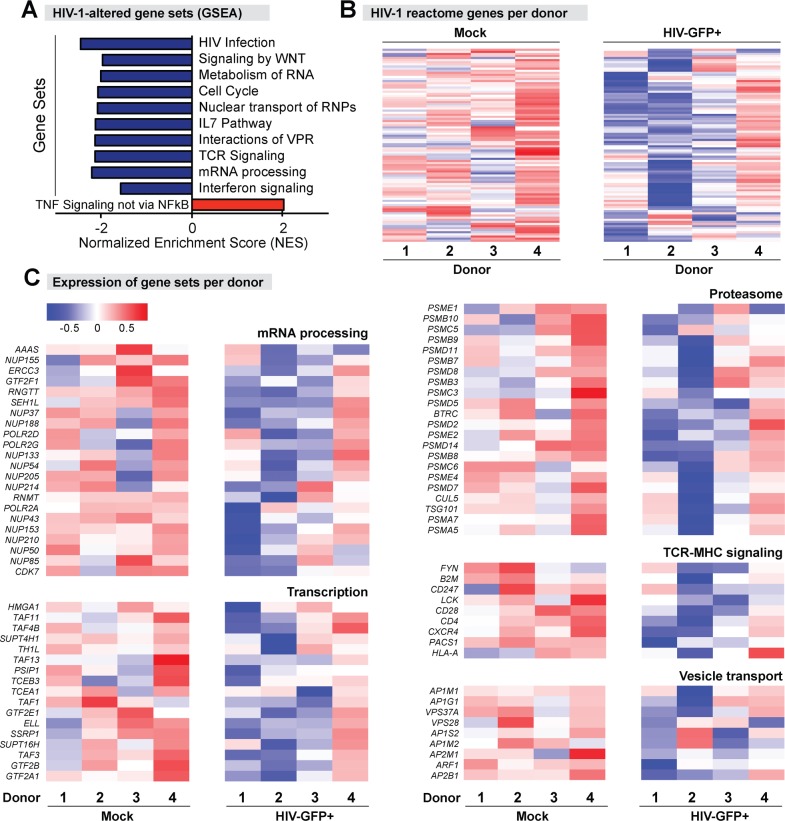
Downregulation of HIV-1 reactome genes in HIV-1-infected gut CD4+ T cells. Gene expression data were subjected to Gene Set Enrichment Analysis (GSEA). For all Panel A, blue bars/panels indicate downregulation, whereas red bars/panels indicate upregulation relative to mock. Color intensities indicate magnitudes of gene expression (log_2_ test/reference). (A) Top-ranked gene sets altered in HIV-1-infected gut CD4+ T cells correspond to HIV-1 reactome gene sets. (B) HIV-1 reactome genes (n = 97) from the GSEA in panel A were extracted and a heat map indicating the relative expression in the 4 different LPMC donors are shown. (C) Downregulation of HIV-1 reactome gene sets following HIV-1 infection. Heat maps of genes associated with mRNA processing, transcription, proteasome, TCR-MHC signaling and vesicle transport in HIV-1 infection are shown for each of the 4 LPMC donors analyzed.

We pre-ranked our entire gene probe list according to t-statistics and then loaded the ranked gene list into GSEA (http://www.broad.mit.edu/gsea/). Positive enrichment scores suggest upregulation of the gene set whereas negative enrichment scores suggest downregulation of the gene set. To gain an overall view of the total number of gene sets enriched, we first investigated canonical pathways. Out of 3,709 canonical pathways, GSEA identified 193 upregulated and 412 downregulated gene sets (normalized or NOM *p*-value <1%) in HIV-eGFP+ cells versus mock. Thus, there was a disproportionate downregulation of canonical pathways in HIV-1-infected cells. Fig 3A outlines some of these gene sets. Notably, the top downregulated gene sets include 97/186 (52%) genes previously associated with HIV-1 infection, which we refer to here as the HIV-1 ‘reactome’ (Fig 3B). A substantial fraction (23/97; 24%) of these HIV-1 reactome genes are associated with mRNA processing, but genes linked to the proteasome, transcription, vesicle transport and TCR-MHC signaling were also downregulated (Fig 3C). Thus, productive HIV-1 infection appears to induce a host cellular ‘shutdown’ response that involves extensive downregulation of host-encoded HIV-1 co-factors.

### HIV-1 perturbs OX40 and PPAR signaling in gut CD4+ T cells

GSEA is a valuable tool for evaluating the enrichment of a defined gene set but is not designed to predict pathway activation or inhibition. By contrast, Ingenuity Pathway Analysis (IPA) combines differential gene expression data with the Ingenuity Pathways Knowledge Base to determine altered canonical pathways, upstream regulators and predicted downstream disease/functions (www.ingenuity.com) [23]. To determine which canonical pathways were activated or inhibited by HIV-1 in gut CD4+ T cells, we performed IPA using a *p*-value cut-off of 0.05 and a fold change cut-off of ±1.25. Positive Z-scores indicate pathway activation whereas negative Z-score suggest pathway inhibition.

**Fig 4:**
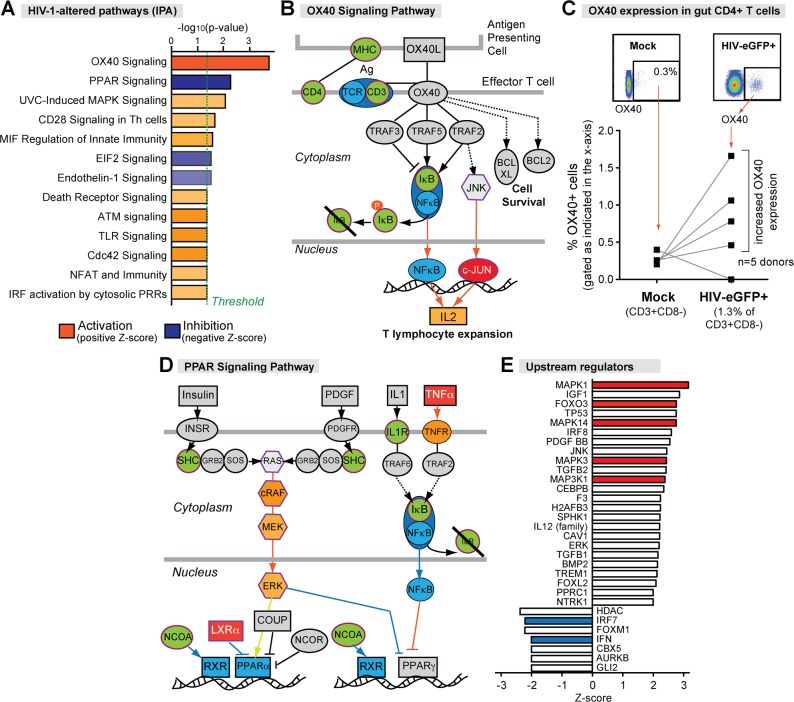
HIV-1 alters multiple signaling pathways in gut CD4+ T cells. Microarray data were subjected to Ingenuity Pathway Analysis (IPA). (A) HIV-1-altered pathways. Bars correspond to log-transformed p-value and colors indicate either pathway activation (orange) or inhibition (blue) based on a Z-score. OX40 and PPAR signaling had the highest Z-scores and *p*-values. (B) OX40 signaling pathway components and (C) evaluation of OX40 expression in gut CD4+ T cells in 5 LPMC donors by flow cytometry. %OX40+ cells were evaluated in total CD4+ T cells from mock-infected cultures versus HIV-1-eGFP+ cells from HIV-1-infected cultures. (D) PPAR signaling pathway components. For panels B and D, non-gray components correspond to relevant genes in the pathway. This includes genes (black border) or complexes (magenta border) that were upregulated (orange-to-red) or downregulated (green). Blue shapes correspond to components of the pathways being investigated. (E) Upstream regulators, either activated (positive Z-score) or inhibited (negative Z-score) based on IPA. Colored bars correspond to selected upstream regulators discussed in the text.

The top canonical pathway induced by HIV-1 in gut CD4+ T cells was OX40 signaling (Z = 2.236; *p =* 0.00018) (Fig 4A). OX40 (CD134) is a member of the TNFR superfamily and is involved in CD4+ T cell regulation [24]. To further delineate the OX40 signaling components altered by HIV-1 infection, we utilized the Molecular Activity Predictor tool in IPA. The OX40 signaling pathway genes altered in HIV-1-infected LP CD4+ T cells include *CD4* (-1.46×), *CD3G* (-1.93×), *HLA-B* (-1.26×), *JUN* (3.06×), *MAPK12* (1.34×) and *NFKB1A* (-2.38×) and the anti-apoptotic factor *Bcl-xL* (1.25×; *p* = 0.058). IPA further predicted that activation of the OX40 signaling by HIV-1 would result in T cell expansion (Fig 4B). To examine if OX40 signaling was altered at the protein level, we evaluated OX40 expression on HIV-1-infected gut CD4+ T cells by flow cytometry. Even though increased *OX40* mRNA was not detected in the microarray, in 4 of 5 LPMC donors, the percentage of OX40+ cells was higher in HIV-1-infected versus mock-infected gut CD4+ T cells (Fig 4C). Altogether, these data suggest that HIV-1 infection may have triggered a pro-survival, OX40-linked pathway in gut CD4+ T cells.

In terms of downregulated pathways, HIV-1 inhibited peroxisome proliferator-activated receptor (PPAR) signaling (Z = -1.90; *p* = 0.0057) in LP CD4+ T cells (Fig 4A). PPARs are nuclear-hormone receptors that in the context of T cell signaling are anti-inflammatory usually through repression of NFkB [25]. The PPAR signaling genes that were altered in HIV-1-infected cells include *CREBBP* (-1.42×), *FOS* (2.49×), *HRAS* (1.37×), *IL18RAP* (2.04×), *JUN* (3.06×), *NFKB1A* (-2.38×), *NR1H3* (1.37×), *NR1P1* (-1.57×), *SHC1* (-1.33×) and *TNF* (2.22×) (Fig 4D). The upregulation of *IL18RAP* and *TNF* in HIV-1-infected gut CD4+ T cells was consistent with an augmented inflammatory signature.

### HIV-1 activates MAPK and FOXO3 transcriptional programs in LP CD4+ T cells

The induction of OX40 signaling at 4 dpi is expected to promote cell survival (Fig 4B and 4C). However, TF HIV-1 also causes CD4+ T cell death by 6 dpi (Fig 1C). Previously, we showed that HIV-1 Ba-L induced CD4+ T cell death in ~50% of LPMC donors at 4 dpi [18]. These data suggested that some gut CD4+ T cells may be poised to undergo death at 4 dpi. To determine if pathways associated with programmed cell death were detectable, we further scanned the IPA results. Indeed, death receptor signaling (*p* = 0.04) was upregulated (Fig 4A). Moreover, DNA damage response pathways such as ‘Radiation (UVC) induced MAPK signaling’ (*p* = 0.008) and ‘Ataxia telangiectasia mutated (ATM) signaling’ (*p* = 0.04) were induced (Fig 4A). ATM signaling is induced in response to chromatin damage due to DNA double strand breaks, a proposed inducer of CD4 T cell death via DNA-PK [26].

To identify upstream master regulators that may promote HIV-1-mediated LP CD4+ T cell death, we utilized IPA's ‘Upstream Regulator Analysis’ tool (Fig 4E). HIV-1 activated MAPK pathways (specifically, MAPK1, MAPK3, MAPK14 and MAP3K1; Z = 2.3 to 3.2; *p*<0.01). MAPK pathways are involved in cell activation, proliferation, differentiation, survival/apoptosis and cytokine production in response to cellular stress response such as DNA damage and viral infection [27]. The HIV-1-altered genes that can be linked to MAPK1 activation include upregulated *FOS*, *JUN*, *TNF*, *ATF3* and *FOSB* as well as downregulated *HLA-B* and interferon-stimulated genes (ISGs) *IFI16*, *IFIT2*, *ISG20* and *OAS3*. Consistent with the downregulation of these ISGs, IPA also predicted the inhibition of upstream regulators IFN and IRF7 (Fig 4D). Interestingly, we identified FOXO3 (Z = 2.760 *p* = 0.002) as an upstream regulator of differentially regulated genes in HIV-1 infected LP CD4+ T cells. FOXO3 is a tumor suppressor gene that can regulate DNA damage responses, as well as CD4+ T cell survival and differentiation [28]. Ten genes were consistent with FOXO3 activation. Of these genes, *TNF* (2.22×), *PPP1R15A* (1.54×), *EGR2* (1.38×) and *EGR1* (4.51×) are pro-apoptotic. Thus, FOXO3 could be functioning as an upstream regulator controlling HIV-1 induced LP CD4+ T cell death.

### Microbial exposure enhanced TF HIV-1 infection and depletion of LP CD4+ T cells

**Fig 5:**
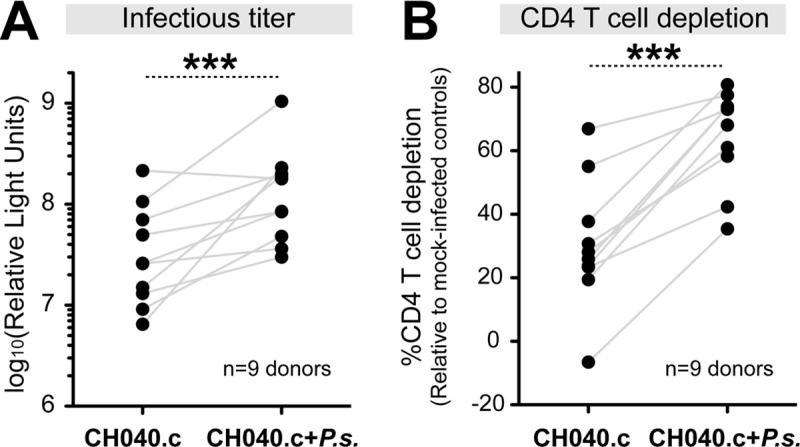
Microbial exposure enhances TF HIV-1 infection and CD4+ T cell death in the LPAC model. After spinoculation with the TF HIV-1 CH040.c strain, LPMCs were resuspended in media containing or not containing heat-killed *Prevotella stercorea* (*P.s.*) at a 2.5 bacteria: 1 LPMC ratio. Supernatants and cells were analyzed at 6 dpi. (A) Infectious titers. Supernatants were evaluated for infectious HIV-1 titers using TZM-bl reporter cells. Log-transformed firefly luciferase values are shown. (B) CD4+ T cell depletion. The difference in the absolute number of CD3+CD8- T cells between HIV-1 infected and uninfected (mock) LPMC cultures were calculated. Mock controls for CH040.c only was not exposed to *P*. *stercorea*, while the mock controls for CH040.c+*P*.*s*. were exposed to *P*. *stercorea*. For both panels, each connected dot corresponds to a different LPMC donor (n = 9 donors). Data were analyzed using a paired 2-tailed Student’s t test. ***p<0.001.

Gut barrier dysfunction during acute HIV-1 infection results in microbial translocation that has been associated with immune activation [29]. Our group and others discovered that bacteria from the *Prevotella* genus were enriched in mucosal tissues of HIV-1-infected individuals [19, 30–34]. Further, *Prevotella* abundance was linked to mucosal mDC and T cell activation [19, 35]. Using the LPAC model, we showed that two representative species, *Prevotella stercorea* and *Prevotella copri*, enhanced HIV-1 Ba-L replication and depletion in intestinal CD4+ T cells [20]. To determine if this phenomenon extends to TF HIV-1 strains, LPMCs were spinoculated with HIV-1 CH040.c, CH058.c and CH470, then incubated in the presence or absence of *Prevotella stercorea*. At 6 dpi, infection levels and CD4+ T cell counts were assessed relative to microbe-exposed mock controls (Fig 1A). Microbial exposure significantly enhanced both HIV-1 CH040.c infection (Fig 5A) and CD4+ T cell killing (Fig 5B). Similar results were also obtained with the CH058.c and CH470 (S5 Fig). CH040.c-eGFP infection was also enhanced by microbial exposure (S1 Fig). These data demonstrate that microbial exposure promotes TF HIV-1 infection and CD4+ T cell depletion in the LPAC model.

### Microbial exposure activated genes and pathways that promote HIV-1 replication

**Fig 6:**
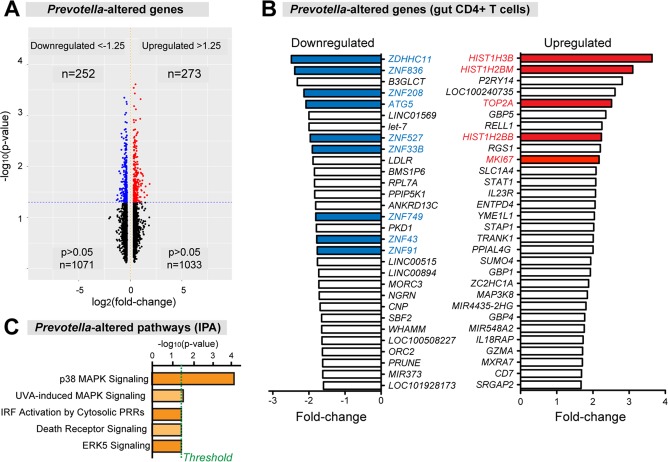
Microbial exposure alters gut CD4+ T cell gene expression. HIV-1-uninfected LPMCs were exposed to mock (media) or *Prevotella stercorea* (2.5 bacteria: 1 LPMC) for 4 days, and sorted gut CD4+ T cells were subjected to microarray analyses. (A) Volcano plots showing differentially regulated genes following microbial exposure. (B) Top 30 ranked down- and upregulated genes in microbe-exposed CD4+ T cells. Colored bars correspond to genes discussed in the text. (C) Ingenuity Pathway Analysis. The pathway with the highest Z-score and p-value is the p38 MAPK pathway.

We next sought to identify transcriptome signals that may explain the increased HIV-1 susceptibility of microbe-exposed LP CD4+ T cells. HIV-1 uninfected LP CD4+ T cells sorted from microbe-untreated and microbe-treated LPMCs (n = 4 donors each) were subjected to microarray analyses. We identified 525 differentially altered genes (Fig 6A). Fig 6B, S6 Fig and S3 Table highlight the top 30 and full list of upregulated and downregulated genes in microbe- versus mock-treated LP CD4+ T cells. Microbial exposure upregulated Ki-67 (2.2×), a marker of cellular proliferation, as well as several histones and DNA topoisomerase. Interestingly, ATG5, a critical component of the autophagy pathway, and multiple zinc finger proteins of unknown function were downregulated (Fig 6B). We next utilized IPA to determine significantly altered pathways; of these, p38 MAPK signaling was significantly induced (Z = 0.707; *p* = 0.00024) (Fig 6C; S6 Fig). This pathway was previously shown to enhance HIV-1 replication in mitogen-activated peripheral blood CD4+ T cells [36, 37].

**Fig 7:**
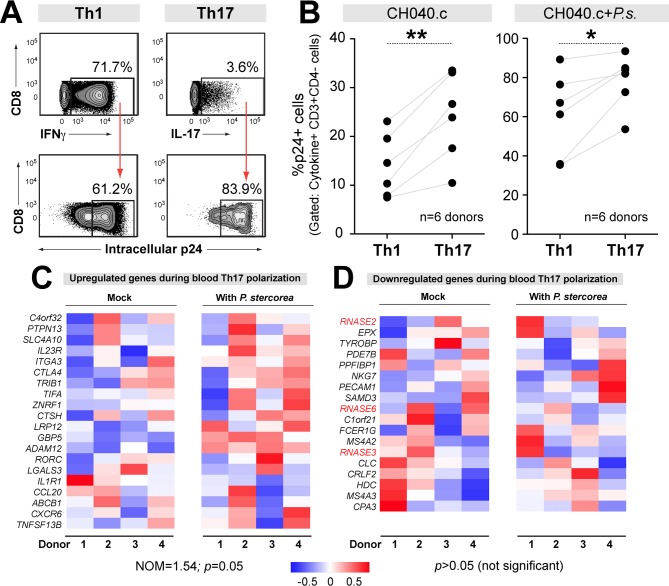
HIV-1 infection of gut Th17 cells. (A-B) Relative TF HIV-1 susceptibility of Th17 versus Th1 cells. LPMCs were infected with HIV-1 CH040.c and (A) the percentage of intracellular p24+ cells were evaluated by flow cytometry in IL17 versus IFNγ producing cells at 4 dpi. (B) Data were analyzed using 2-tailed paired Student’s t test; **, *p*<0.01; *, *p*<0.05. (C-D) Expression of Th17 differentiation genes. Polarization of blood Th0 cells into Th17 cells was associated with (C) gene upregulation (n = 20 genes) and (D) gene downregulation (n = 18 genes) [42]. These gene lists were extracted from [42] and subjected to GSEA. (C) Upregulated Th17 genes were induced (NOM = 1.54, *p* = 0.05) in microbe-exposed gut CD4+ T cells. (D) By contrast, downregulated Th17 genes, including RNAses 2, 3 and 6 (in red), were not significantly perturbed (*p*>0.05). Higher color intensities indicate higher magnitudes of expression changes.

CD4+ T cells are classified into different subsets depending on the predominant cytokines they produce. Our group and others reported that IL17-producing CD4+ T cells (Th17), and in particular, those producing IFNγ and IL17 (Th1/17), were highly susceptible to HIV-1 infection *in vitro* [18, 38–40]. Th1/17 cells appeared to have a higher state of cellular activation and lower antiviral properties based on transcriptional profiling [41]. To extend these findings to TF HIV-1, we evaluated the relative susceptibility of gut Th1 versus Th17 cells by p24 flow cytometry (Fig 7A). Regardless of microbial exposure, we observed higher percentages of TF HIV-1-infected Th17 versus Th1 cells (Fig 7B). A recent microarray study revealed 38 differentially altered genes in mitogen-activated, peripheral blood CD4+ T cells grown under Th17 polarizing conditions (S4 Table) [42]. The authors linked the downregulation of *RNASE2*, *RNASE3* and *RNASE6* to enhanced susceptibility of Th17 cells HIV-1 infection [42]. We tested if gene sets linked to ‘Th17 susceptibility’ were similarly altered in microbe-exposed gut CD4+ T cells. Indeed, using GSEA, upregulated Th17 co-factor genes were similarly altered in microbe-exposed LP CD4+ T cells (Fig 7C). However, *RNASE2*, *RNASE3* and *RNASE6* expression were not inhibited in microbe-exposed CD4+ T cells (Fig 7D). Overall, our findings suggest that the increased susceptibility of microbe-exposed LP CD4+ T cells to HIV-1 may be due to a combination of factors that include increased cellular proliferation, p38 MAPK activation, induction of host co-factors required for viral DNA replication and enhanced Th17 cell differentiation.

### Microbial exposure stimulated a strong interferon signature in LP CD4+ T cells

**Fig 8:**
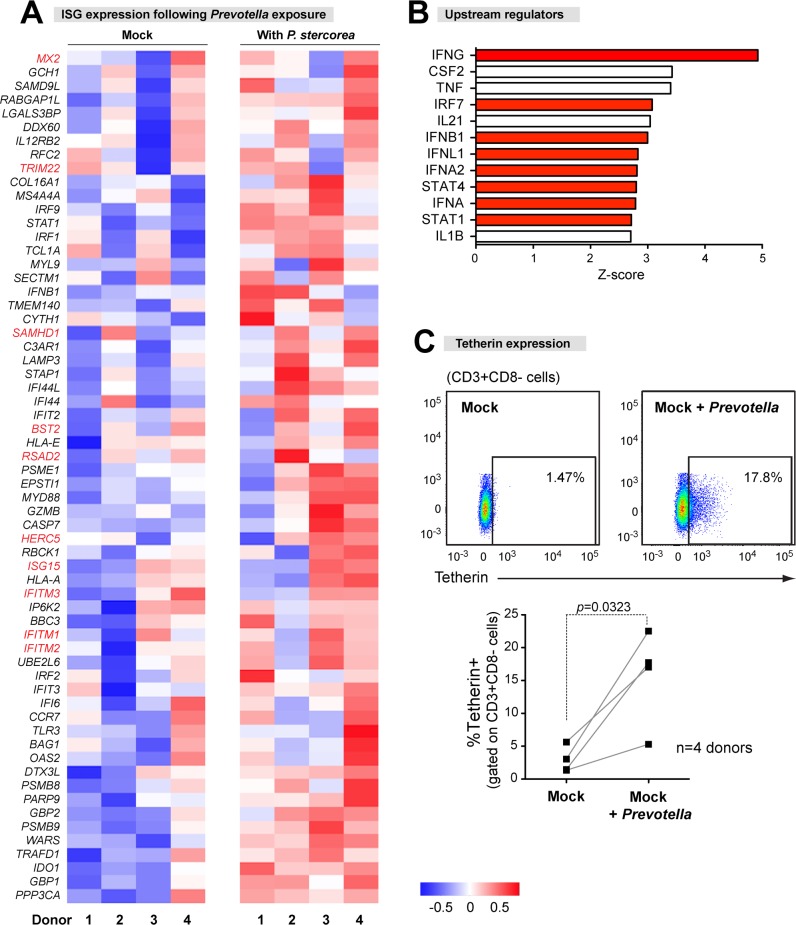
Microbial exposure induces a robust type I IFN signature in gut CD4+ T cells. (A) ISG expression. Gene set enrichment analyses (GSEA) predicted the upregulation of multiple ISGs in microbe-exposed gut CD4+ T cells. These 62 genes were extracted to generate a heat map detailing individual LPMC donors. Blue bars/panels indicate downregulation, whereas red bars/panels indicate upregulation relative to mock. Higher color intensities indicate magnitudes of gene expression (log_2_ test/reference values). Genes highlighted in red are known retrovirus restriction factors. (B) IPA-predicted upstream regulators. Red bars correspond to upstream regulators involved in IFN signaling that were discussed in the text. (C) Microbial induction of tetherin/BST-2 on gut CD4+ T cells, as measured by flow cytometry. A representative flow cytometry plot is shown. Data were analyzed using paired 2-tailed Student’s t-test, *, *p*<0.05.

HIV-1 can be inhibited by restriction factors, a collective term for host-encoded proteins with direct antiretroviral activity. Thus, we hypothesized that in addition to the upregulation of host co-factors, the increased susceptibility of microbe-exposed LP CD4+ T cells to HIV-1 may be due to the downregulation of restriction factors. Most antiviral restriction factors are induced by type I interferons (IFN). Surprisingly, GSEA found that 6 of the 8 top gene sets enriched in microbe-exposed LP CD4+ T cells were Type I interferon-responsive genes. Fig 8A highlights the extensive upregulation of GSEA-compiled ISGs (n = 62) in microbe-exposed gut CD4+ T cells. This included several known retroviral restriction factors such as *MX2*, *SAMHD1*, *Tetherin (BST2)*, *Viperin (RSAD2)*, *ISG15* and *IFITMs* [43] (S5 Table). Furthermore, using IPA, the upstream regulators of differentially-expressed genes included IRF7 (*p* = 0.00025), IFNβ1 (*p* = 0.0046), IFNα2 (*p* = 0.00007) and IFNα (*p* = 0.00081), as well as sole type II IFN, IFNG (*p =* 0.000052) and a type III IFN, IFNλ1 (*p* = 0.000069) (Fig 8B). ISG induction at the protein level was confirmed by analyzing tetherin/BST2 expression on microbe-exposed gut CD4+ T cells from 4 LPMC donors (Fig 8C). Thus, microbial exposure stimulated with a strong IFN gene signature in gut CD4+ T cells.

### Differential effects of HIV-1 infection on microbe-exposed LP CD4+ T cells

**Fig 9:**
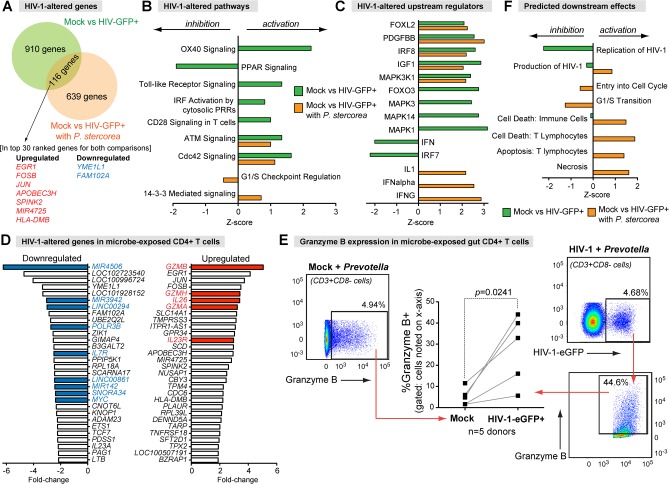
HIV-1 infection differentially alters multiple pathways in microbe-exposed gut CD4+ T cells. *Prevotella stercorea* were utilized as representative enteric bacteria found in the mucosa of HIV-1-infected individuals. (A) Overlap in HIV-1-altered genes between gut CD4+ T cells exposed or not exposed to bacteria. (B) Comparison of HIV-1-altered pathways with or without microbial exposure using Ingenuity Pathway Analyses (IPA). (C) Comparison of HIV-1-altered upstream regulators based on IPA. (D) HIV-1-altered genes in microbe-exposed CD4+ T cells. Colored bars were discussed in the text. (E) Induction of Granzyme B in HIV-1-infected, microbe-exposed gut CD4+ T cells. The percentages of granzyme B+ cells were evaluated in CD3+CD8- cells from mock-infected LPMCs exposed to *Prevotella stercorea* versus HIV-1-eGFP+ cells from HIV-1-infected LPMCs exposed to *Prevotella stercorea*. Data were analyzed using a paired 2-tailed Student’s t-test. (F) IPA-predicted downstream effects, based on differentially altered genes.

We showed in Figs 3 and 4 that HIV-1 infection induced OX40 and FOXO3 signaling while downregulating HIV-1-reactome and PPAR signaling in LP CD4+ T cells. Since microbial exposure triggered significant transcriptome changes, we next investigated if HIV-1 infection altered the same gene sets/pathways in microbe-exposed versus non-microbe exposed LP CD4+ T cells. LPMCs were either mock- or HIV-1-infected, then incubated with *P*. *stercorea*. At 4 dpi, HIV-1-GFP+CD3+CD8- cells and counterpart microbe-exposed mock controls were sorted for microarray analyses. Notably, only a modest overlap (15%, Fig 9A) was observed between the genes that were differentially-expressed due to HIV-1 infection in the presence or absence of microbial exposure. The genes altered by HIV-1 independent of microbial exposure included *EGR1*, *JUN*, *FOSB*, *APOBEC3H* and *FAM102A* (Fig 9A), which were also reported in HIV-1 infected blood CD4+ T cells (Fig 1B) [21]. In contrast, *HLA-DMB*, *MIR4725*, *SPINK2* and *YME1L1* were altered in HIV-1-infected gut CD4+ T cells regardless of bacterial exposure, but these genes were not altered by HIV-1 in blood CD4+ T cells [21]. Of note, HLA-DMB is a peptide exchange factor involved in MHC class II antigen presentation, an operational process in human CD4+ T cells [44].

IPA provided insights on the microbe-independent pathways altered by HIV-1 infection. HIV-1 activated Cdc42 and ATM signaling (Fig 9B), as well as upstream regulators MAPK3K1, IGF1, IRF8, PDGFBB and FOXL2 with or without microbial exposure (Fig 9C). By contrast, HIV-1 failed to downregulate HIV-1-reactome genes based on GSEA (NOM *p*>0.05) and failed to perturb OX40, PPAR, MAPK1/3/14 and FOXO3 signaling based on IPA in microbe-exposed LP CD4+ T cells (Fig 9B and 9C). Microbial exposure also differentially altered the impact of HIV-1 on upstream IFN regulators (Fig 9C). As shown earlier, microbial exposure alone already induced multiple ISGs (Fig 8; S7 Fig and S5 Table). HIV-1 further induced 17% of these ISGs in microbe-exposed CD4+ T cells including *BST2*, *IFI44L*, *IFIT3*, *ISG15*, *PARP9* and *RSAD2*, as well as 32 additional ISGs (S5 Table). This contrasts from our data showing HIV-1-mediated downregulation of ISGs in the absence of microbial exposure (Fig 4E). Thus, microbial exposure significantly influenced how HIV-1 altered the transcriptome gut CD4+ T cells.

### HIV-1 infection may alter the function of microbe-exposed LP CD4+ T cells

We next inspected the top-ranked genes altered by HIV-1 in microbe-exposed CD4+ T cells (Fig 9D, S8 Fig and S6 Table). Several microRNAs were inhibited, possibly linked to RNA polymerase III (*POLR3B*) downregulation (Fig 9D). Interestingly, HIV-1 infection triggered a significant increase in granzyme A, B and H expression in LP CD4+ T cells, which we confirmed at the protein level by flow cytometry (Fig 9E). Granzymes are typically produced by cytotoxic CD8+ T cells and natural killer cells, where they function as apoptosis-inducing serine esterases [45]. Only a small subset of CD4+ T cells express granzymes, and these CD4+ T cells exhibit cytotoxic properties [46]. These findings suggested that HIV-1 infection may have conferred cytotoxic properties to microbe-exposed LP CD4+ T cells. Furthermore, 2 genes linked to Th17 function were significantly induced. HIV-1 infection of microbe-exposed LP CD4+ T cells stimulated 3.4-fold higher levels of *IL26*, a Th17-derived cytokine that has antibacterial properties [47], and 3.0-fold higher levels of the *IL23R*, which acts as the receptor of IL23 and helps maintain Th17 cell function [48]. Thus, HIV-1 infection may have enhanced the antibacterial properties of microbe-exposed LP CD4+ T cells.

Intriguingly, the IPA results suggested that HIV-1 may have perturbed cell cycle processes in microbe-exposed CD4+ T cells. The 14-3-3 pathway is activated; this pathway is critical for cell cycle progression (Fig 9B) [49]. Moreover, genes associated to the G1/S checkpoint regulation, particularly *MYC*, were inhibited in HIV-1-infected LP CD4+ T cells exposed to microbes (Fig 9D, S8 Fig) [50]. Downregulation of c-Myc can in turn inhibit CDK4/6 and cyclin D activation (S9 Fig) [51]. As noted earlier, HIV-1 infection further enhanced the type I IFN response (Fig 9C, S5 Table). Type I IFNs can block the cell cycle at the G1 phase [52]. These findings suggest that productive HIV-1 infection may cause cell cycle arrest at the G1/S phase in microbe-exposed CD4+ T cells.

### Increased cell death signals in HIV-1-infected, microbe-exposed LP CD4+ T cells

In the absence of microbial exposure, we detected apoptosis signals in HIV-1-infected LP CD4+ T cells (FOXO3), but these were counterbalanced by survival signals (OX40). To predict potential outcomes from gene expression data, ‘downstream analysis’ can be performed through IPA. Using IPA's knowledge base of differential gene expression in varying disease and functional states, a *p*-value for overlap can be calculated by Fisher’s exact test. An activation Z-score determines if there was a significant pattern match between the predicted and observed up/downregulation for each disease/function analyzed.

Downstream analysis revealed that the gene expression profile of HIV-1-infected, microbe-exposed LP CD4+ T cells predicted a strong infection, cell cycle perturbation and cell death outcome (Fig 9F). IPA predicted cell death of immune cells, cell death of T lymphocytes, apoptosis and necrosis based on specific genes that were altered (S7 Table). By contrast, these disease/function states were not significantly predicted in LP CD4+ T cells infected with HIV-1 in the absence of microbial exposure (Fig 9F). Thus, microbial exposure appears to have increased the potential of HIV-1 to directly cause cell death.

## Discussion

Acute HIV-1 infection causes profound CD4+ T cell loss in the GI tract, and the sequelae of dysbiosis, barrier dysfunction, microbial translocation and chronic inflammation likely contributes to various renal, cardiac, liver, vascular and pulmonary co-morbidities that does not resolve with antiretroviral therapy [14, 53]. Documenting the transcriptome of gut CD4+ T cells infected with HIV-1 may thus provide insights on mucosal HIV-1 pathogenesis. Previous studies showed that HIV-1 altered the transcriptome of CD4+ T cell lines and mitogen-activated peripheral blood CD4+ T cells [2, 21, 54]. Given that HIV-1 infection in culture is quite low (<5% GFP+ cells), cell lines and blood CD4+ T cells are advantageous because large numbers of these cells can be infected. By contrast, obtaining large numbers of CD4+ T cells from tissues for sorting productively infected, HIV-1-GFP+ cells is logistically difficult. Here, we obtained large numbers of LPMCs from 4 donors that allowed us to profile the transcriptome of TF HIV-1-infected gut CD4+ T cells for the first time. The limited RNA yields, particularly for HIV-1 GFP+ cells, were just enough to perform microarray analyses. Microarrays have limited dynamic range and may underestimate fold-differences in gene expression, compared to more sensitive techniques such as real-time PCR [55]. To circumvent this limitation, rather than solely highlighting individual gene changes, we focused our analysis on the coordinate regulation of multiple genes and/or gene sets linked to a certain pathway or phenotype.

We initially compared our set of differentially altered genes to published microarray results on HIV-1-infected CD4+ T cell lines and mitogen-activated peripheral blood CD4+ T cells. We found at least 2 results in common with HIV-1-infected gut CD4+ T cells. First, HIV-1 induced *JUN* and *FOS* gene expression [21, 56, 57]. JUN and FOS form the AP1 transcription factor [58]. Multiple AP1 binding sites are found in the HIV-1 LTR, which can drive HIV-1 viral transcription [59]. Thus, AP1 may be critical in driving productive HIV-1 replication in gut CD4+ T cells. Second, HIV-1 infection of gut CD4+ T cells downregulated HIV-1-reactome (cofactor) genes at 4 dpi, mirroring data on HIV-1 infection of SupT1 cell lines at 1 dpi [54]. These findings suggest a sustained host cellular shutdown response to infection that may impose blocks for HIV-1 superinfection. Efforts are ongoing to genetically inactivate individual viral genes in TF HIV-1 to determine the viral mechanisms driving the HIV-1-induced host cellular shutdown response in primary gut CD4+ T cells.

We should emphasize that the similarities between blood versus gut gene expression profiles were rare. Differences in the Th cell subset composition in the blood (naïve/central memory) versus the gut (effector memory) may explain the lack of overlap between differentially expressed genes, but naïve and memory CD4+ T cells share 95% of their transcriptomes [60]. We expected that if genes shared between blood and gut CD4+ T cells were essential for replication, these genes should have been similarly altered by HIV-1. Instead, >97% of the genes that HIV-1 altered in gut CD4+ T cells were not detected in mitogen-activated peripheral CD4+ T cells. One explanation is that the selection criteria used to identify altered genes in HIV-1-infected gut CD4+ T cells (this study) was different from that of the blood [21] to accommodate the heterogeneity of LPMC donors. However, despite our less stringent fold-change criteria, we still did not capture a substantial fraction of HIV-1-altered genes reported in blood CD4+ T cells.

We postulate that several features of the LPAC model that distinguish it from standard HIV-1 infection of PBMCs may have contributed to the differences in gene expression between blood and gut CD4+ T cells. Standard HIV-1 infection of PBMCs requires exogenous mitogens, which maximize HIV-1 infection and minimize donor-to-donor variation in HIV-1 replication. While this method has technical advantages particularly for *in vitro* HIV-1 propagation, exogenous mitogen activation of PBMCs may mask subtle effects of HIV-1 on CD4+ T cell activation and function. By contrast, LPMCs do not require exogenous mitogens for HIV-1 infection. LPMCs also contain myeloid DCs (mDCs) that have distinct phenotypic properties compared to those found in blood [61]. The presence of mDCs may explain the strong induction of OX40 signaling in HIV-1-infected gut CD4+ T cells, as OX40 induction requires its ligand in antigen-presenting cells, OX40L. Of note, OX40-OX40L signaling promoted the survival of memory CD4+ T cells within the gut lamina propria [62], and enhanced productive HIV-1 infection in activated blood CD4+ T cells [63]. Our data implied the existence of viral mechanisms that promote the survival of the infected cell, presumably to enhance virus production.

In the LPAC model, TF HIV-1 depleted gut CD4+ T cells starting at 6 dpi. Thus, the induction of a pro-survival pathway (OX40) at 4 dpi (the time point we used for microarray analyses) may be followed by the induction of cell death pathways. Multiple MAP kinases and FOXO3, a transcription factor involved in apoptosis induction in part through the induction of DNA damage responses [64, 65], were predicted as upstream regulators in infected gut CD4+ T cells at 4 dpi. These data suggest that there may be a subset of gut CD4+ T cells at 4 dpi that were poised to undergo apoptotic cell death. The mRNA used for transcriptomic profiling came from bulk CD4+ T cells, making it difficult to determine if survival and death mechanisms were simultaneously operating in an HIV-1-infected cell. Single-cell transcriptomics should provide more in-depth information on the transition from survival to death during the course of HIV-1 infection in the LPAC model. In addition to the activation of survival and death pathways in HIV-1-infected gut CD4+ T cells, PPAR signaling, an anti-inflammatory pathway [25], was inhibited. Two genes inhibited by PPAR signaling, *TNFα* and *IL18RAP*, were induced. TNF*α* is a proinflammatory cytokine that can induce apoptosis [66]. IL18RAP is part of the heterodimeric receptor for the proinflammatory cytokine IL18, which can also induce apoptosis. Interestingly, *IL18RAP* polymorphisms have been associated with inflammatory bowel disease [67]. Further studies would be required to track the kinetics of OX40, FOXO3, MAPK and PPAR signaling in mediating CD4+ T cell survival, death and inflammation.

Gut barrier disruption occurs a few weeks after HIV-1 infection, resulting in microbial translocation. Bacteria from the *Prevotella* genus may be important, as these bacteria were abundant in the mucosal tissues of HIV-1-infected individuals, either as a result of HIV-1 infection itself or sexual practices [68]. Moreover, mucosa-associated *Prevotella* abundances correlated with mucosal T cell and DC activation *in vivo* [19, 35]. Using the LPAC model, we found that these enteric microbes promoted HIV-1 replication and gut CD4+ T cell depletion. Enhanced HIV-1 replication was linked to increased CCR5 expression, activation and proliferation of gut CD4+ T cells [20], but the underlying molecular pathways that regulate these processes remain unclear. Thus, a major goal of the current study is to determine how microbial exposure changes the CD4+ T cell transcriptome. In addition to gene signatures indicative of proliferation (Ki67) and activation (p38 MAPK), we detected an increase in HIV-1 ‘dependency factors’ that were recently linked to Th17 polarization [42]. Previously, we showed that HIV-1 infection of LPMCs *ex vivo* is skewed towards Th17, Th1 and particularly Th1/17 cells [17, 18]. Here we extended these data, showing that gut Th17 cells supported higher TF HIV-1 infection levels than Th1 cells. Of note, co-incubation of LPMCs with *E*. *coli* enhanced Th17 proliferation [17]. Our data are consistent with an increase in more HIV-1 susceptible Th17 cells in microbe-exposed LPMCs.

The enhanced TF HIV-1 susceptibility of microbe-exposed gut CD4+ T cells may also be a consequence of downregulated antiviral genes. Surprisingly, we observed a strong type I IFN signature in microbe-exposed gut CD4+ T cells. ISGs encoding potent antiretroviral restriction factors such as tetherin/BST-2 were induced following microbial exposure. Despite the induction of multiple antiviral genes, microbial exposure enhanced TF HIV-1 replication. Microbial exposure also induced genes associated with T cell proliferation and activation, suggesting that microbial exposure may have raised the threshold for innate antiviral factors to confer protection. In other words, the induction of restriction factors was overcome by the enhanced expression of HIV-1 susceptibility genes in microbe-exposed gut CD4+ T cells. Of note, early HIV-1 infection in the gut was associated with increased type I IFN responses [16]. Our data suggest that the gut CD4+ T cell response to microbes may contribute to the enhanced type I IFN responses in mucosal tissues of individuals with early HIV-1 infection.

One important finding from the current study is that microbial exposure influenced how HIV-1 altered the gut CD4+ T cell transcriptome. HIV-1 did not perturb the OX40, PPAR, FOXO3 and HIV-1-reactome genes in microbe-exposed CD4+ T cells, in contrast to what we observed in the absence of microbial exposure. Instead, HIV-1 appears to have augmented the antibacterial properties of microbe-exposed gut CD4+ T cells, based on the upregulation of granzymes, *IL26* and *IL23R*. We speculate that these phenotypic changes may perpetuate and exacerbate inflammation in the GI tract. For example, the release of granzymes by cytotoxic CD4+ T cells, especially in the context of potentially increased MHC class II presentation by HIV-1-infected CD4+ T cells, may further disrupt the epithelial barrier. Notably, the gene expression profile in HIV-1-infected gut CD4+ T cells strongly predicted apoptotic cell death. This prediction is consistent with our previous report showing enhanced HIV-1-mediated apoptotic CD4+ T cell death with microbial exposure [18]. Disruptions in the cell cycle, particularly at the G1/S phase, were hinted by the gene expression data. These findings are in agreement with the strong type I IFN signature of microbe-exposed CD4+ T cells, which was enhanced even further by HIV-1 infection. Type I IFN signaling can suppress cellular growth and promote apoptosis through the inhibition of CDK4/6, cyclins and c-Myc, which were observed in our study. Our findings suggest a model in which CD4+ T cell-intrinsic type I IFN signaling due to microbial exposure may potentiate gut CD4+ T cells for accelerated HIV-1 mediated death.

In conclusion, we provide the first transcriptome study of TF HIV-1-infected gut CD4+ T cells in the presence or absence of enteric bacteria *ex vivo*. Our findings confirm and/or identify novel HIV-1- and microbe-induced molecular pathways that may regulate HIV-1 infection, CD4+ T cell depletion, and immune dysregulation inside and outside the context of microbial translocation in the intestinal mucosa. Further validation and in-depth mechanistic studies on these pathways in the LPAC model, and *in vivo* through human clinical studies and the SIV/rhesus macaque model, should provide more basic insight on mucosal HIV-1 pathogenesis. Such investigations may unravel novel therapeutic targets to counteract HIV-1 infection and inflammation in the GI tract.

## Materials and methods

### Ethics statement

Jejunum samples that would otherwise be discarded were obtained from HIV-1 uninfected patients undergoing elective surgery at the University of Colorado Hospital. These patients signed a release form for the unrestricted use of the tissues for research purposes following de-identification to laboratory personnel. The procedures were approved and given exempt status by the Colorado Multiple Institutional Research Board at the University of Colorado.

### Plasmid and bacterial stocks

We previously reported on the generation of Transmitted/Founder (TF) HIV-1 infectious molecular clones (IMCs) CH040.c and CH058.c [4]. TF IMC CH470 was generously provided by Beatrice Hahn (U. Pennsylvania) [69]. pCH040.c-GFP.T2A/K3223 (referred to herein as CH040.c-eGFP) was constructed from pCH040.c (GenBank #JN944939.1) by inserting eGFP using the *nef* ATG, followed by a self-cleaving T2A peptide cassette and *nef* in frame, analogous to a previous approach [70]. pCH470, pCH040.c and pCH058.c plasmids were prepared using Stbl3 cells (Invitrogen, Carlsbad, CA) and used to produce viral stocks in 293T cells as previously described [71]. Virus stocks were titered using an HIV-1 Gag p24 ELISA kit (Perkin Elmer, Waltham, Massachutsetts). *Prevotella stercorea* (DSM #18206, DSMZ, Braunschweig, Germany) stocks were prepared as previously detailed [35], frozen at -80°C in DPBS in single use aliquots and enumerated using CountBright counting beads and a LSRII flow cytometer (BD Bioscience, San Jose, CA).

### Human Lamina Propria Aggregate Culture (LPAC) and HIV-1 infection

Human jejunal tissue samples were obtained from the patients undergoing elective surgery and disaggregated as previously described [17, 18, 72] and cryopreserved until use. Lamina propria mononuclear cells (LPMCs) were thawed using a standard protocol as detailed previously [18] and resuspended in RPMI, 10% human AB serum (Gemini Bioproducts, West Sacramento, CA), 1% penicillin/streptomycin/glutamine (Life Technologies, Grand Island, NY), 500 μg/ml Zosyn (piperacillin/tazobactium; Wyeth, Madison, NY) (cRPMI). 500 ng p24 of TF IMCs CH040.c, CH058.c or CH470 or the GFP reporter TF IMC, CH040.c-eGFP, were spinoculated into 2.5 × 10^6^ LPMCs. For microarray analyses, CH040.c.-eGFP infections were performed on six replicate tubes containing 2.5 × 10^6^ LPMCs (total of 15 million cells). LPMCs were mock infected in parallel. Infection by spinoculation was performed at 1500 × *g* for 2 h at room temperature. After 2 h of spinoculation, supernatant containing the free virus was discarded and the LPMCs were washed with cRPMI. LPMCs were resuspended at 10^6^ LPMCs/ml in cRPMI and plated into a 96-well V-bottom culture dish and live *P*. *stercorea* was added (2.5 *P*. *stercorea* per 1 LPMC) where appropriate. LPMCs were cultured for 4 or 6 days at 37°C, 5% CO_2_ and 95% humidity.

### Quantification of Infectious virus and CD4+ T cell depletion

LPMCs were infected with normalized input of TF HIV-1 stocks as described above (‘LPAC culture’) and input virus was removed after 2 h. Infectious virus production from supernatants harvested at 6 dpi from LPMCs was measured in TZM-bl reporter cells [73] (NIH AIDS Reagent Program #8129) as previously described [71]. For this, TZM-bl cells were plated in an opaque flat bottom 96-well plate; HIV-1 containing supernatants were added in 10-fold dilutions, mixed and incubated for 48 hours at 37°C. After 48 hours, cells were lysed using Britelite luciferase reagent (Perkin Elmer, Waltham, MA) and Relative Light Units (RLU) of luminescence were determined on a VictorX5 plate reader (Perkin Elmer, Waltham, MA). To quantify depletion, the difference in the number of viable T cells in HIV-1-infected cultures was compared to the number of viable T cells in matched mock-infected cultures. This ratio was reported as the percentage of T cells depleted from the HIV-1-infected wells as previously described [18]. All conditions were run in triplicate wells with the average depletion for the 3 wells reported.

### Flow cytometry

To determine percentages of p24+ cytokine+ cells, LPMC were collected at 4dpi and stimulated with 250 ng/ml PMA (Sigma-Aldrich) and 1 μg/ml ionomycin (Sigma-Aldrich) in the presence of 1 μg/ml brefeldin A (Golgi Plug; BD Biosciences, San Jose, CA) for 4 h at 37°C, 5% CO_2_. Percentages of IFNγ and IL17-producing CD4 T cells expressing p24 were determined by intracellular flow cytometry as previously described [17, 18].

To validate certain altered genes/pathways from the microarray analyses, LPMCs (from donors different from those used in the microarray) were infected with CH040.c-eGFP or mock and stimulated with or without *P*. *stercorea* as detailed above. At 4 dpi, total LPMCs were collected and stained with PerCP-Cy5.5 α-CD45 (2D1, eBioscience, San Diego, CA), Zombie Aqua Fixable Viability dye (Biolegend, San Diego, CA), PE α-CD3 (OKT3, Tonbo Biosciences, San Diego, CA), PE-Cy7 α-CD8 (RPA-T8, Tonbo Biosciences), APC-Cy7 α-OX40 (CD134; Ber-ACT35, Biolegend), APC α-tetherin (CD317; RS38E, Biolegend) following standard flow cytometry protocols [17, 18, 20]. Cells were fixed (Medium A; Life Technologies), permeabilized (Medium B; Life Technologies) and stained with Pacific Blue α-Granzyme B (GB11; Biolegend) following standard intracellular staining flow cytometry protocols [17, 18, 20]. Manufacturer recommend isotype controls were used to establish OX40, tetherin and Granzyme B-specific staining. Data acquisition was performed using an LSRII flow cytometer (BD Biosciences) and analysis performed using FlowJo Software (V10).

### Separation of HIV-1 infected and uninfected bystander cells

After 4 d in culture, cells were surface-stained with PerCP-Cy5.5 α-CD3 (OKT3, Tonbo Biosciences), APC α-CD8 (OKT8, Tonbo Biosciences), and Zombie Viability Dye (Biolegend). HIV-1 productively infected cells were sorted using a Moflo Astrios EQ cell sorter based on live CD3+CD8- GFP+ staining. Mock infected, *P*. *stercorea* exposed and non-productively HIV-1 infected cells were sorted based on live CD3+CD8-GFP- staining.

### Microarray and bioinformatics analysis

Total RNA from sorted fresh cell pellets was isolated using the RNAeasy micro kit according to the manufacturers instructions (QIAGEN, Valencia, CA). The integrity of extracted RNA from sorted cells was analyzed using an Agilent Tapestation 2200 and concentrations were determined using qubit 2.0 fluorometer (Thermo Fisher Scientific) instrument. Transcriptional analysis was done at the University of Colorado Denver Genomics and Microarray Core facility. Following the manufacturer’s protocol, starting total RNA was converted to cDNA with the GeneChip WT Pico Kit (Affymetrix) and amplified for 13 PCR cycles. *In vitro* transcription was performed using the same kit to generate cRNA. The cRNA was converted into single-strand cDNA in a second cycle. The cDNA was fragmented and end-labeled with biotin. After standard labeling, each sample was hybridized to a GeneChip Human Gene 2.0 ST array, followed by examination of fluorescence intensity of the probes with an Affymetrix GeneChip Scanner 3000 7G.

Statistical analyses were performed using the open-source R (version 2.11) statistical software with supporting statistical libraries from the Bioconductor consortium (www.bioconductor.org) [74]. Raw data were transformed using the Robust Multi-chip Average (RMA) normalization method for background correction [75]. Principal Component Analyses (PCA) were plotted in 3D using the R library rgl (https://cran.r-project.org/web/packages/rgl/vignettes/rgl.html). Given the heterogeneity of LPMC samples by PCA (S3A Fig), false discovery rates [76] were >1%. Differentially-expressed genes consistent between the 4 LPMC donors were identified based on paired Student’s t-test with *p*<0.05 and a fold-change cut-off of 1.25 (S3B Fig).

### Gene set enrichment analysis

Gene set enrichment analysis (GSEA) was performed using an open-source software package (version 2.0, Broad Institute http://www.broad.mit.edu/gsea) [22]. First, a ranked list was obtained by performing paired-t test between groupings of interest. The ranked list was ordered based on the paired-t statistics ranging from the most positive to the most negative. Then the association between a given gene set and the group was measured by the non-parametric running sum statistic termed the enrichment score (ES). To estimate the statistical significance of the ES, a nominal (NOM) *p* value was calculated by permuting the genes 1,000 times. To adjust for multiple hypotheses testing, the maximum ES was normalized to account for the gene set size (NES) and the false discovery rate (FDR). The gene sets used were from Molecular Signatures Database (MsigDB), catalog C2 (version 3.0) functional sets, subcatalog canonical pathways, which include 880 gene sets from pathway databases. These gene sets were collected from online databases such as Bio-Carta, Reactome, and KEGG (Kyoto Encyclopedia of Genes and Genomes).

### Ingenuity Pathway Analysis

Ingenuity Pathway Analysis (IPA) software (Ingenuity Systems, www.ingenuity.com) was used to identify canonical signaling pathways, upstream regulators and downstream disease/function pathways associated with the expression profiles of HIV-1 GFP+ vs. Mock, *P*. *stercorea* vs. Mock, *P*. *stercorea* HIV-1 GFP+ vs. *P*. *stercorea*. Differentially expressed Affymetrix Probe IDs were imported into the Ingenuity software and mapped to the Gene Symbol from the Ingenuity knowledge database. A *p*-value cutoff of <0.05 and fold change cutoff of ± 1.25 was used to filter the differentially expressed gene list into IPA. The significance of the association between the dataset and the canonical pathway, upstream regulator, downstream disease/function was determined by over-representation. Fisher's exact test was used to calculate a *p*-value determining the probability that the association between the genes in the dataset and the canonical pathway, upstream regulator or downstream disease/functions could be explained by chance alone. A *p*-value <0.05 was used as the cut-off for identifying significant altered canonical pathways, upstream regulators or downstream disease/functions. Z-scores were calculated by IPA to infer predicted canonical pathways, upstream regulators, disease/function activation or inhibition. Briefly, the basis for inferences are edges (relationships) in the molecular network that represent experimentally observed gene expression or transcription events, and are associated with a literature‐derived regulation direction which can be either “activating” or “inhibiting” [23]. Positive Z-scores indicate activation whereas negative Z-scores indicate inhibition.

### Statistical analysis

Statistical analyses were performed using GraphPad Prism version 6 for Windows (GraphPad Software, San Diego, CA). Data were analyzed using a 2-tailed Student’s t test (either paired or unpaired). For multiple comparisons, 1-way ANOVA followed by a Dunnett’s posthoc test was used. P values <0.05 were considered significant.

### Accession number

Microarray data from this study was deposited in the NCBI Gene Expression Omnibus (GEO) Database Accession GSE86404.

## Supporting information

S1 Fig Expression of HIV-1 p24 in HIV-1-eGFP+ cells. Viable CD3+CD8- cells infected with HIV-1 CH040.c-eGFP were evaluated for eGFP and intracellular p24 expression at 4 dpi by flow cytometry. Data are presented for LPMCs exposed or not exposed to *Prevotella stercorea* from 3 different donors. Donor numbers were designated 5–7 to distinguish them from the donors used for microarray analyses (donors 1–4). Note that a fraction of HIV-1-eGFP-negative cells express low levels of p24. These eGFP^neg^p24^low^ cells may harbor productive infection, abortive infection or virus particles just sticking on the surface of the CD4+ T cells. Thus, HIV-1-eGFP^neg^ cells may be a mixture of HIV-1-infected, ‘bystander’, and uninfected cells.
(TIF)

S2 Fig Gating strategy for sorting viable HIV-1-infected gut CD4+ T cells. Since HIV-1 can downregulate CD4, infected CD4+ T cells were sorted as CD3+CD8- cells. Up to 15 million LPMCs were sorted to obtain sufficient numbers of HIV-1-infected (GFP+) CD4+ T cells for microarray analyses.
(TIF)

S3 Fig Criteria for differentially-expressed genes. (A) Principal Component Analysis of log_2_-transformed gene expression data from 4 LPMC donors at 4 dpi. The six experimental conditions evaluated per LPMC donor include: mock without *Prevotella*, HIV-1-eGFP+ without *Prevotella*, HIV-1-eGFP^neg^ without *Prevotella*, mock with *Prevotella*, HIV-1-eGFP+ with *Prevotella* and HIV-1-eGFP^neg^ with *Prevotella*. (B) Identification of differentially-regulated genes. Sample data comparing genes from mock with *Prevotella* versus HIV-eGFP+ with *Prevotella*. (1) Log_2_-transformed test/reference values were evaluated per donor. Genes consistently upregulated or downregulated in 4 of 4 LPMC donors are shown in green and red, respectively. (2) Paired 2-tailed Student’s t-test was used to calculate *p*-values. At a *p*<0.05 cut-off, even genes with consistent patterns across the 4 donors (e.g., *MIR1180* and *AFG3L2*) were excluded from the list. (3) Significantly altered genes were further subjected to a 1.25-fold change cutoff, where fold change = 2^mean(HIVPS minus mockPS). In this case, *RIPK4* was excluded from the list.
(TIF)

S4 Fig Heatmaps of top 30 differentially-expressed genes in HIV-1-infected CD4+ T cells relative to mock. Color intensities were based on log_2_(test/reference) data.
(TIF)

S5 Fig Microbial exposure enhances TF HIV-1 infection and CD4+ T cell death in the LPAC model. After spinoculation with the TF HIV-1 CH058.c and CH470 strains, LPMCs were resuspended in media containing or not containing heat-killed *Prevotella stercorea* at a 2.5 bacteria: 1 LPMC ratio. Supernatants and cells were analyzed at 6 dpi. (A) Infectious titers. Supernatants were evaluated for infectious HIV-1 titers using the TZM.bl assay. Log-transformed luciferase values are shown. (B) CD4+ T cell depletion. The difference in the absolute number of CD4+ T cells between HIV-1 infected and uninfected (mock) LPMC cultures were calculated. Mock controls for TF HIV-1 only was not exposed to *P*. *stercorea*, while the mock controls for TF HIV-1+*P*.*s*. were exposed to *P*. *stercorea*. For both panels, each connected dot corresponds to a different LPMC donor (n = 9–10 donors). Data were analyzed using a paired 2-tailed Student’s t test. ***p<0.001.
(TIF)

S6 Fig Activation of the p38 MAPK pathway in microbe-exposed gut CD4+ T cells. Microarray data were subjected to Ingenuity Pathway Analysis (IPA). Non-gray components correspond to relevant genes in the pathway. This includes genes (black border) or complexes (magenta border) that were upregulated (orange-to-red) or downregulated (green). Blue shapes correspond to components of the pathways being investigated.
(TIF)

S7 Fig Heatmaps of top 30 differentially-expressed genes in gut CD4+ T cells exposed to *Prevotella stercorea*. Color intensities were based on log_2_(test/reference) data.
(TIF)

S8 Fig Heatmaps of top 30 differentially-expressed genes in HIV-1-infected, microbe-exposed CD4+ T cells. Color intensities were based on log_2_(test/reference) data.
(TIF)

S9 Fig G1/S cell cycle perturbation in HIV-1-infected gut CD4+ T cells exposed to *Prevotella stercorea*. Microarray data were subjected to Ingenuity Pathway Analysis (IPA). Non-gray components correspond to relevant genes in the pathway. This includes genes (black border) or complexes (magenta border) that were upregulated (orange-to-red) or downregulated (green). Blue shapes correspond to components of the pathways being investigated.
(TIF)

S1 Table Genes similarly altered by HIV-1 in gut and mitogen-activated blood CD4+ T cells. (XLSX)

S2 Table Upregulated and downregulated genes in gut CD4+ T cells following HIV-1 infection. (XLSX)

S3 Table Upregulated and downregulated genes in gut CD4+ T cells following exposure to *Prevotella stercorea*. (XLSX)

S4 Table Genes associated with peripheral blood Th17 cell polarization. (XLSX)

S5 Table Microbe-induced ISGs *versus* HIV-1-induced ISGs in microbe-exposed gut CD4+ T cells. (XLSX)

S6 Table Upregulated and downregulated genes in microbe-exposed gut CD4+ T cells following HIV-1 infection. (XLSX)

S7 Table Gene expression changes that served as the basis for predicted downstream effects of HIV-1 infection. (XLSX)
