# Supplementary figures and images for: The transcriptome of HIV-1 infected intestinal CD4+ T cells exposed to enteric bacteria
Source: PLoS Pathog. 2017 Feb 27;13(2):e1006226. doi: 10.1371/journal.ppat.1006226 (PMC5344538; doi:10.1371/journal.ppat.1006226)

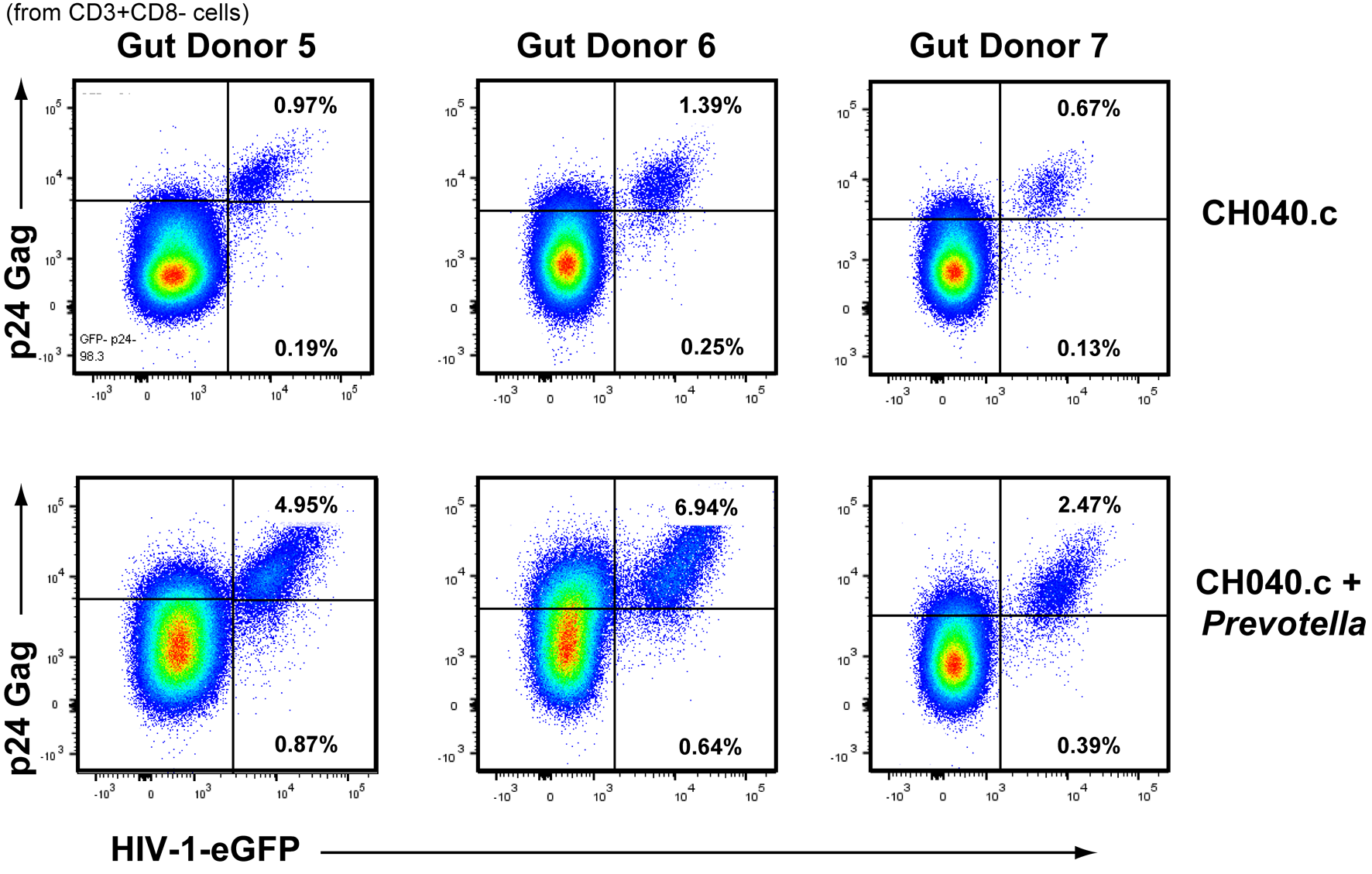

Supplement: S1 Fig — Viable CD3+CD8- cells infected with HIV-1 CH040.c-eGFP were evaluated for eGFP and intracellular p24 expression at 4 dpi by flow cytometry. Data are presented for LPMCs exposed or not exposed to Prevotella stercorea from 3 different donors. Donor numbers were designated 5–7 to distinguish them from the donors used for microarray analyses (donors 1–4). Note that a fraction of HIV-1-eGFP-negative cells express low levels of p24. These eGFPnegp24low cells may harbor productive infection, abortive infection or virus particles just sticking on the surface of the CD4+ T cells. Thus, HIV-1-eGFPneg cells may be a mixture of HIV-1-infected, ‘bystander’, and uninfected cells. (TIF) [file ppat.1006226.s001.tif]

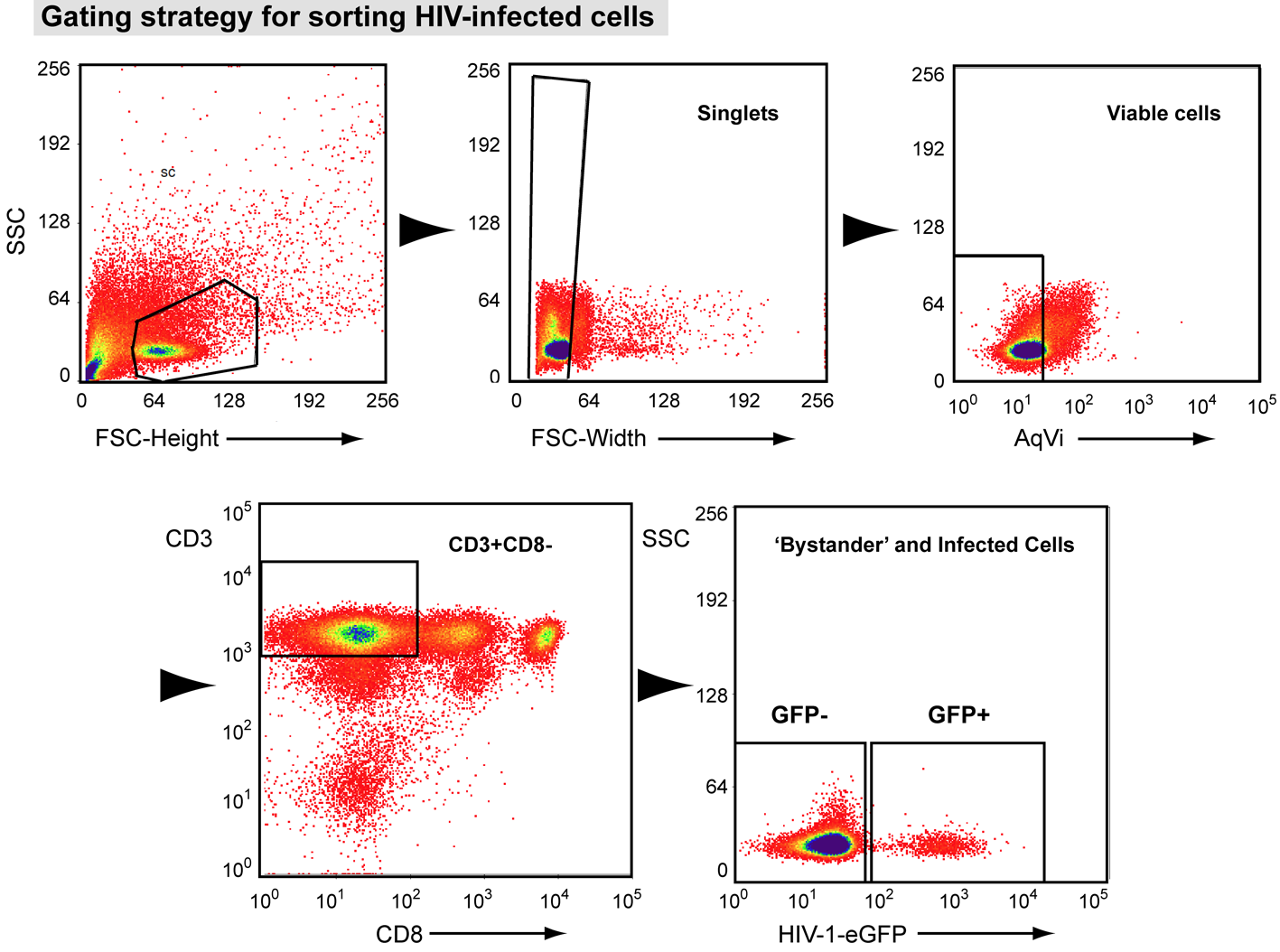

Supplement: S2 Fig — Since HIV-1 can downregulate CD4, infected CD4+ T cells were sorted as CD3+CD8- cells. Up to 15 million LPMCs were sorted to obtain sufficient numbers of HIV-1-infected (GFP+) CD4+ T cells for microarray analyses. (TIF) [file ppat.1006226.s002.tif]

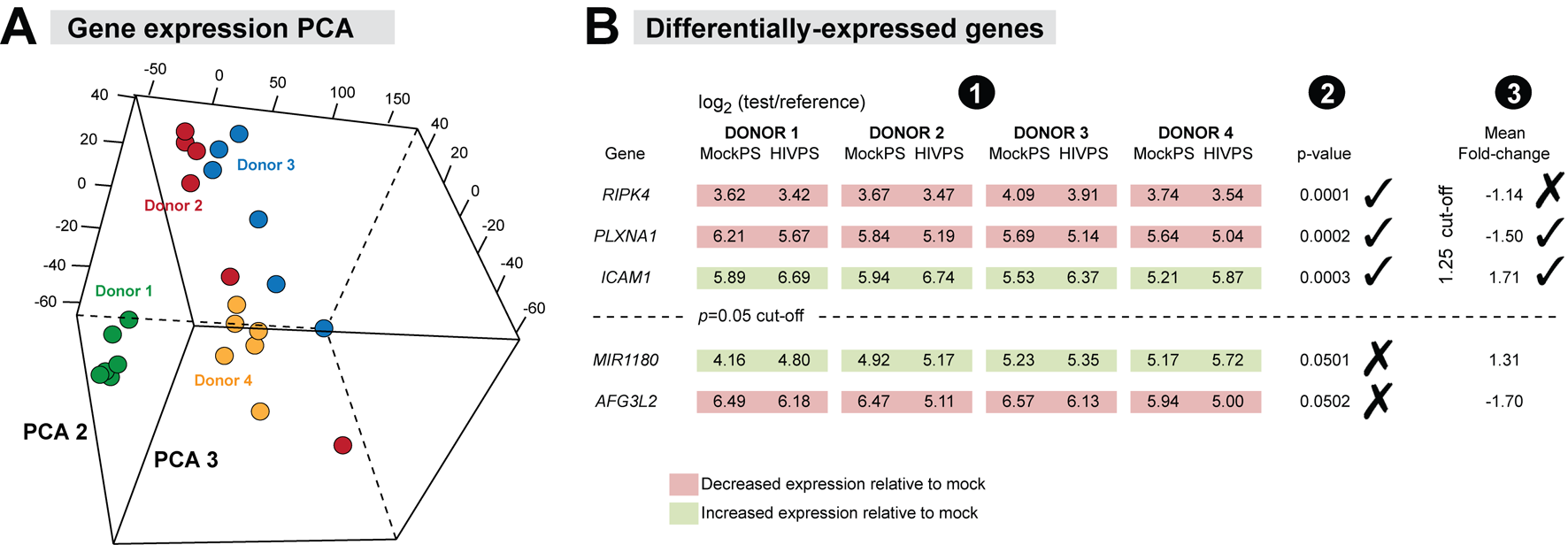

Supplement: S3 Fig — (A) Principal Component Analysis of log2-transformed gene expression data from 4 LPMC donors at 4 dpi. The six experimental conditions evaluated per LPMC donor include: mock without Prevotella, HIV-1-eGFP+ without Prevotella, HIV-1-eGFPneg without Prevotella, mock with Prevotella, HIV-1-eGFP+ with Prevotella and HIV-1-eGFPneg with Prevotella. (B) Identification of differentially-regulated genes. Sample data comparing genes from mock with Prevotella versus HIV-eGFP+ with Prevotella. (1) Log2-transformed test/reference values were evaluated per donor. Genes consistently upregulated or downregulated in 4 of 4 LPMC donors are shown in green and red, respectively. (2) Paired 2-tailed Student’s t-test was used to calculate p-values. At a p<0.05 cut-off, even genes with consistent patterns across the 4 donors (e.g., MIR1180 and AFG3L2) were excluded from the list. (3) Significantly altered genes were further subjected to a 1.25-fold change cutoff, where fold change = 2^mean(HIVPS minus mockPS). In this case, RIPK4 was excluded from the list. (TIF) [file ppat.1006226.s003.tif]

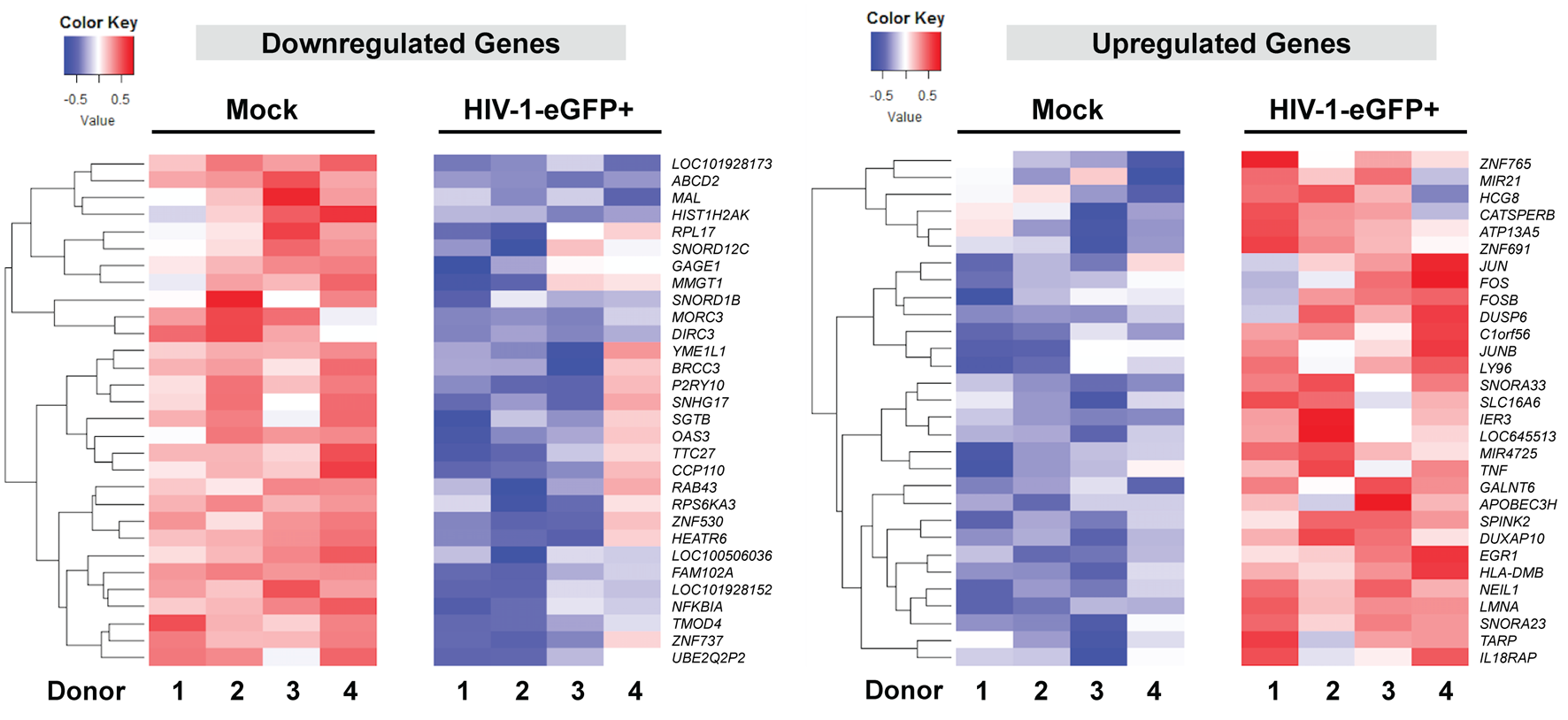

Supplement: S4 Fig — Color intensities were based on log2(test/reference) data. (TIF) [file ppat.1006226.s004.tif]

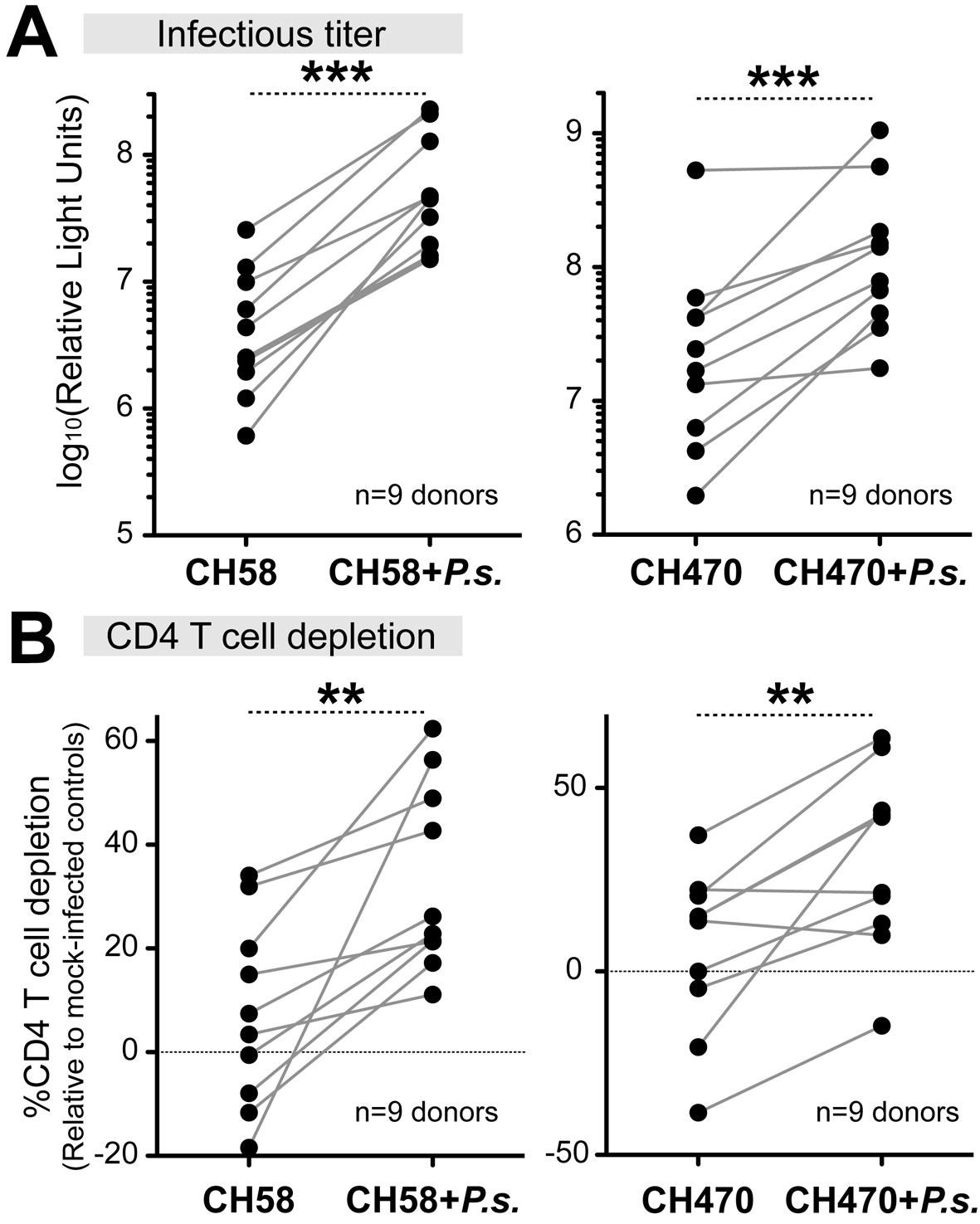

Supplement: S5 Fig — After spinoculation with the TF HIV-1 CH058.c and CH470 strains, LPMCs were resuspended in media containing or not containing heat-killed Prevotella stercorea at a 2.5 bacteria: 1 LPMC ratio. Supernatants and cells were analyzed at 6 dpi. (A) Infectious titers. Supernatants were evaluated for infectious HIV-1 titers using the TZM.bl assay. Log-transformed luciferase values are shown. (B) CD4+ T cell depletion. The difference in the absolute number of CD4+ T cells between HIV-1 infected and uninfected (mock) LPMC cultures were calculated. Mock controls for TF HIV-1 only was not exposed to P. stercorea, while the mock controls for TF HIV-1+P.s. were exposed to P. stercorea. For both panels, each connected dot corresponds to a different LPMC donor (n = 9–10 donors). Data were analyzed using a paired 2-tailed Student’s t test. ***p<0.001. (TIF) [file ppat.1006226.s005.tif]

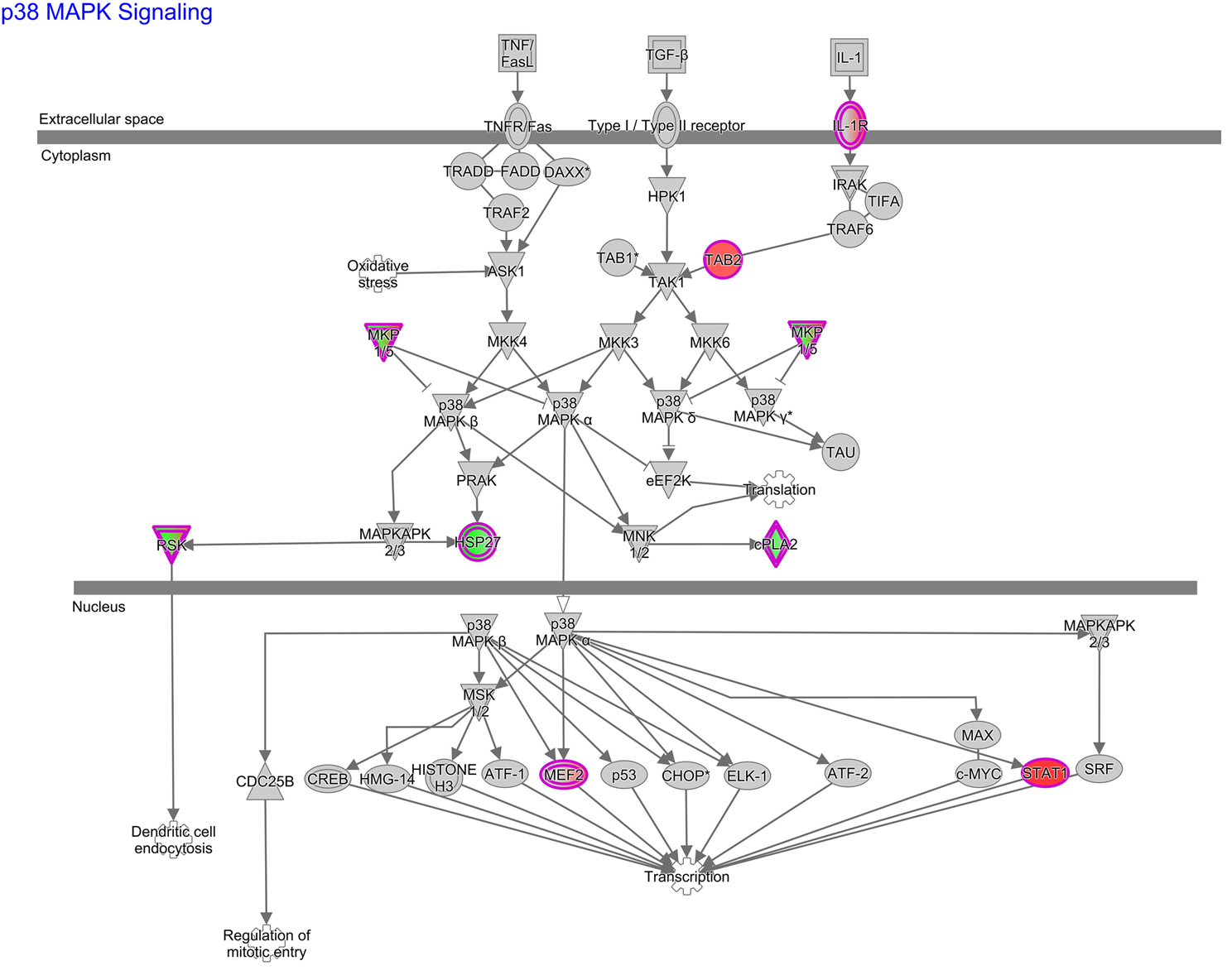

Supplement: S6 Fig — Microarray data were subjected to Ingenuity Pathway Analysis (IPA). Non-gray components correspond to relevant genes in the pathway. This includes genes (black border) or complexes (magenta border) that were upregulated (orange-to-red) or downregulated (green). Blue shapes correspond to components of the pathways being investigated. (TIF) [file ppat.1006226.s006.tif]

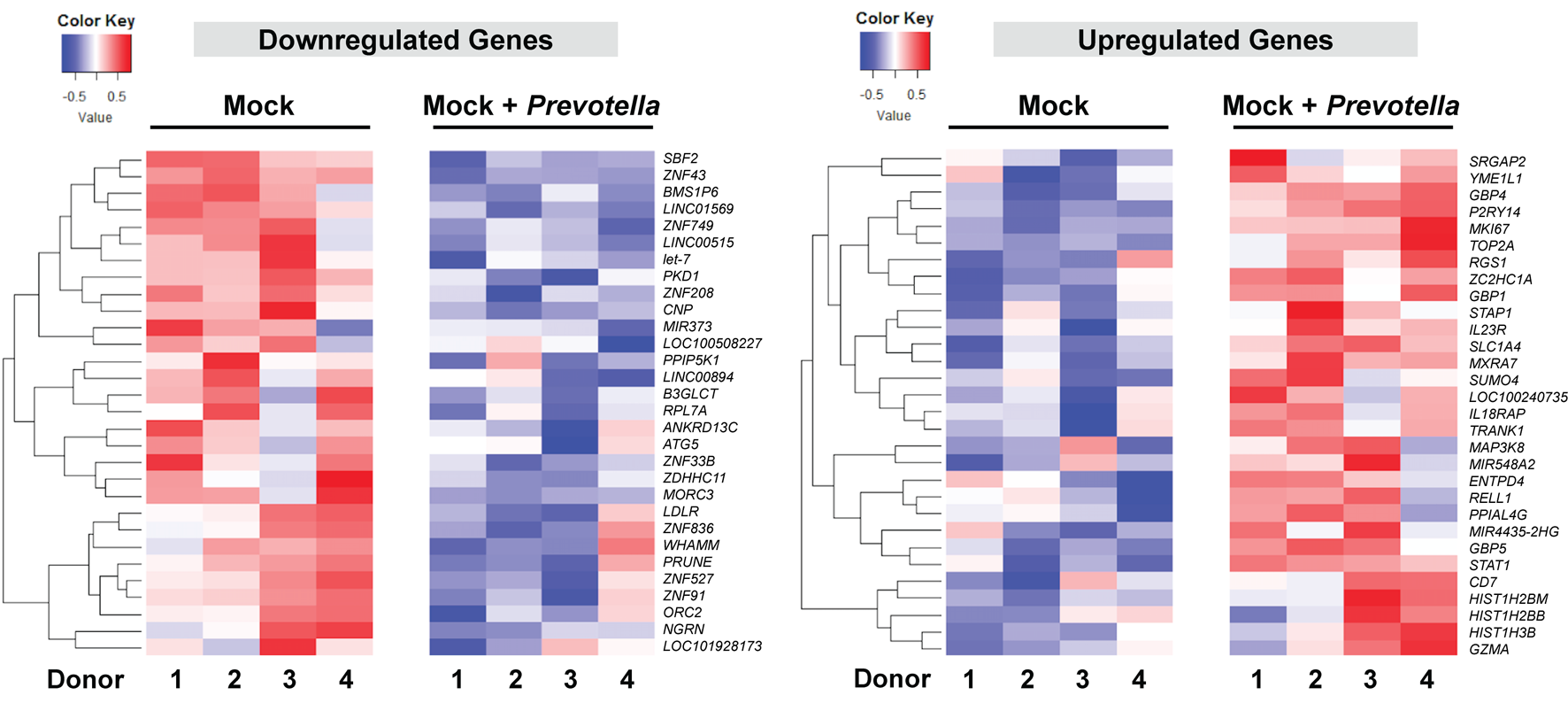

Supplement: S7 Fig — Color intensities were based on log2(test/reference) data. (TIF) [file ppat.1006226.s007.tif]

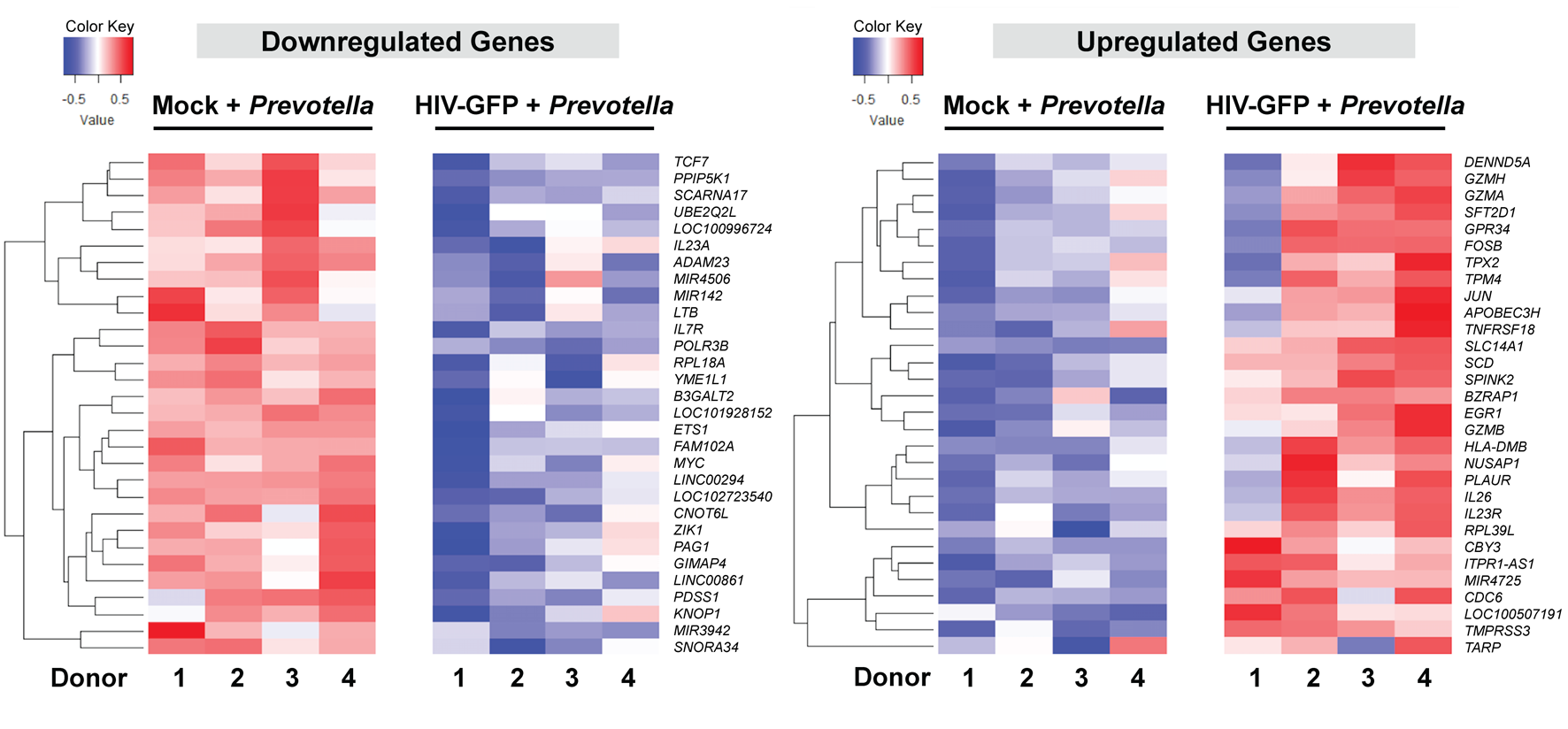

Supplement: S8 Fig — Color intensities were based on log2(test/reference) data. (TIF) [file ppat.1006226.s008.tif]

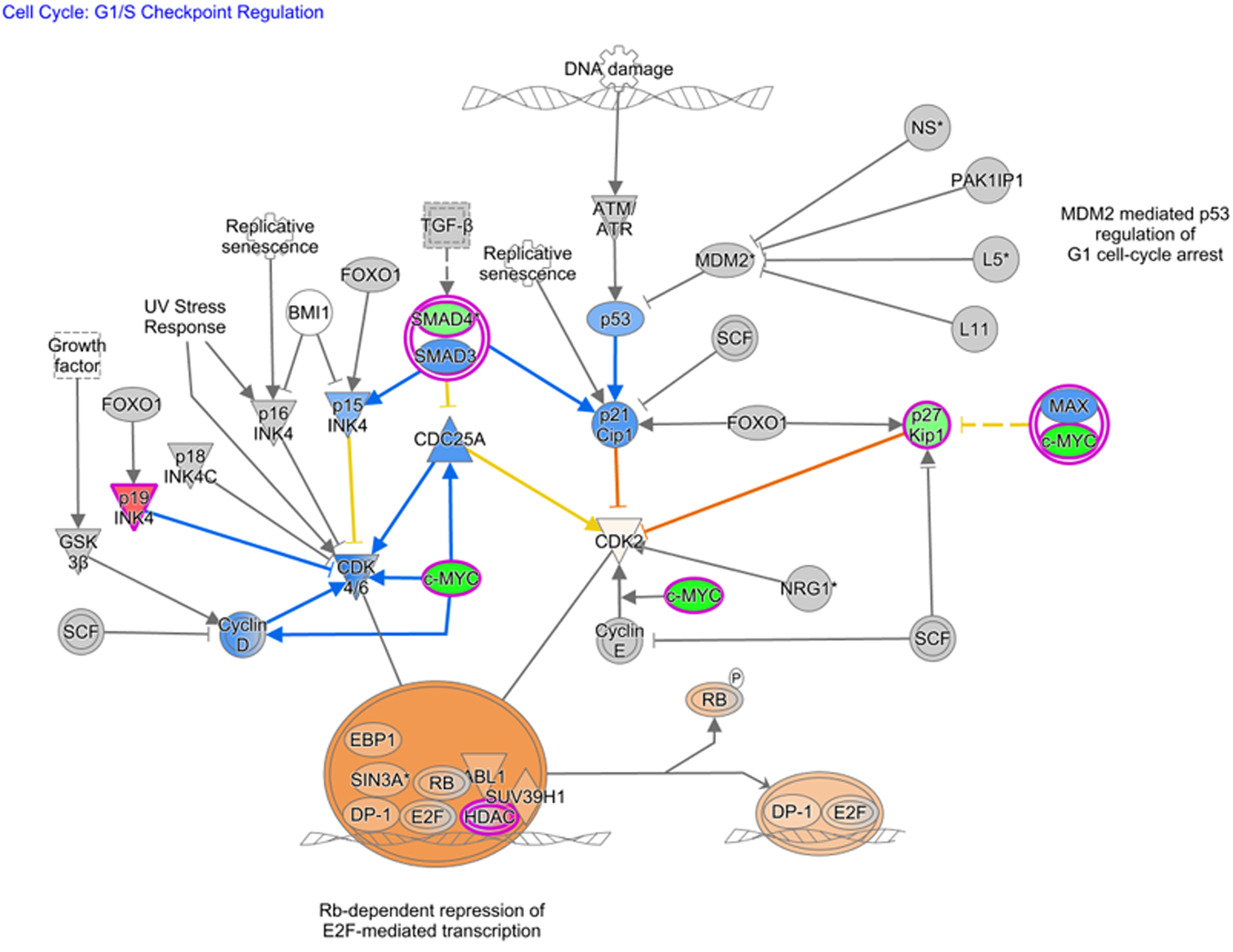

Supplement: S9 Fig — Microarray data were subjected to Ingenuity Pathway Analysis (IPA). Non-gray components correspond to relevant genes in the pathway. This includes genes (black border) or complexes (magenta border) that were upregulated (orange-to-red) or downregulated (green). Blue shapes correspond to components of the pathways being investigated. (TIF) [file ppat.1006226.s009.tif]
